# Genomic Analysis of the Yet-Uncultured Binatota Reveals Broad Methylotrophic, Alkane-Degradation, and Pigment Production Capacities

**DOI:** 10.1128/mBio.00985-21

**Published:** 2021-05-18

**Authors:** Chelsea L. Murphy, Andriy Sheremet, Peter F. Dunfield, John R. Spear, Ramunas Stepanauskas, Tanja Woyke, Mostafa S. Elshahed, Noha H. Youssef

**Affiliations:** a Department of Microbiology and Molecular Genetics, Oklahoma State University, Stillwater, Oklahoma, USA; b Department of Biological Sciences, University of Calgary, Calgary, Alberta, Canada; c Civil and Environmental Engineering, Colorado School of Mines, Golden, Colorado, USA; d Bigelow Laboratory for Ocean Sciences, East Boothbay, Maine, USA; e Department of Energy Joint Genome Institute, Berkley, California, USA; University of Washington

**Keywords:** Binatota, comparative genomics, environmental genomics, metagenomics, phylogenomics

## Abstract

The recent leveraging of genome-resolved metagenomics has generated an enormous number of genomes from novel uncultured microbial lineages yet left many clades undescribed. Here, we present a global analysis of genomes belonging to Binatota (UBP10), a globally distributed, yet-uncharacterized bacterial phylum. All orders in Binatota encoded the capacity for aerobic methylotrophy using methanol, methylamine, sulfomethanes, and chloromethanes as the substrates. Methylotrophy in Binatota was characterized by order-specific substrate degradation preferences, as well as extensive metabolic versatility, i.e., the utilization of diverse sets of genes, pathways, and combinations to achieve a specific metabolic goal. The genomes also encoded multiple alkane hydroxylases and monooxygenases, potentially enabling growth on a wide range of alkanes and fatty acids. Pigmentation is inferred from a complete pathway for carotenoids (lycopene, β- and γ-carotenes, xanthins, chlorobactenes, and spheroidenes) production. Further, the majority of genes involved in bacteriochlorophyll *a*, *c*, and *d* biosynthesis were identified, although absence of key genes and failure to identify a photosynthetic reaction center preclude proposing phototrophic capacities. Analysis of 16S rRNA databases showed the preferences of Binatota to terrestrial and freshwater ecosystems, hydrocarbon-rich habitats, and sponges, supporting their potential role in mitigating methanol and methane emissions, breakdown of alkanes, and their association with sponges. Our results expand the lists of methylotrophic, aerobic alkane-degrading, and pigment-producing lineages. We also highlight the consistent encountering of incomplete biosynthetic pathways in microbial genomes, a phenomenon necessitating careful assessment when assigning putative functions based on a set-threshold of pathway completion.

## INTRODUCTION

Approaches that directly recover genomes from environmental samples, e.g., single-cell genomics and genome-resolved metagenomics, and hence bypass the hurdle of cultivation have come of age in the last decade. The resulting availability of environmentally sourced genomes, obtained as SAGs (single amplified genomes) or MAGs (metagenome-assembled genomes), is having a lasting impact on the field of microbial ecology. Distinct strategies are employed for the analysis of the deluge of obtained genomes. Site- or habitat-specific studies focus on spatiotemporal sampling of a single site or habitat of interest. Function-based studies focus on genomes from single or multiple habitats to identify and characterize organisms involved in a specific process, e.g., cellulose degradation (1) or sulfate reduction (2). Phylogeny-oriented (phylocentric) studies, on the other hand, focus on characterizing genomes belonging to a specific lineage of interest. The aim of such studies is to delineate pan, core, and dispensable gene repertoires for a target lineage, document the lineage’s defining metabolic capabilities (3, 4), understand the lineage’s putative roles in various habitats (5, 6), and elucidate genomic basis underpinning the observed niche specializing patterns (7). The scope of phylocentric studies could range from the analysis of a single genome from a single ecosystem (8) to global sampling and *in silico* analysis efforts (9, 10). The feasibility and value of phylocentric strategies have recently been enhanced by the development of a genome-based (phylogenomic) taxonomic outline based on extractable data from MAGs and SAGs providing a solid framework for knowledge building and data communication (11), as well as recent efforts for massive, high-throughput binning of genomes from global collections of publicly available metagenomes in GenBank nr and Integrated Microbial Genomes & Microbiomes (IMG/M) database (12, 13). As such, these studies provide immensely useful information on potential metabolic capabilities and physiological preferences of yet-uncultured taxa. However, such *in silico* predictions require confirmation through enrichment, isolation, complementary cloning, and expression studies of the gene of interest or other functional genomics approaches to ascertain their phenotypic relevance.

Candidate phylum UBP10 has originally been described as one of the novel lineages recovered from a massive binning effort that reconstructed thousands of genomes from publicly available metagenomic data sets (12). UBP10 has subsequently been named candidate phylum Binatota (henceforth Binatota) in an effort to promote nomenclature for uncultured lineages based on attributes identified in MAGs and SAGs (14). The recent generation of 52,515 distinct MAGs binned from over 10,000 metagenomes (13) has greatly increased the number of available Binatota genomes. Here, we utilize a phylocentric approach and present a comparative analysis of the putative metabolic and biosynthetic capacities and putative ecological roles of members of the candidate phylum Binatota, as based on sequence data from 108 MAGs. Our study documents aerobic methylotrophy, aerobic alkane degradation, and carotenoid pigmentation as defining traits in the Binatota. We also highlight the presence of incomplete chlorophyll biosynthetic pathways in all genomes and propose several evolutionary-grounded scenarios that could explain such a pattern.

## RESULTS

### Genomes analyzed in this study.

**FIG 1:**
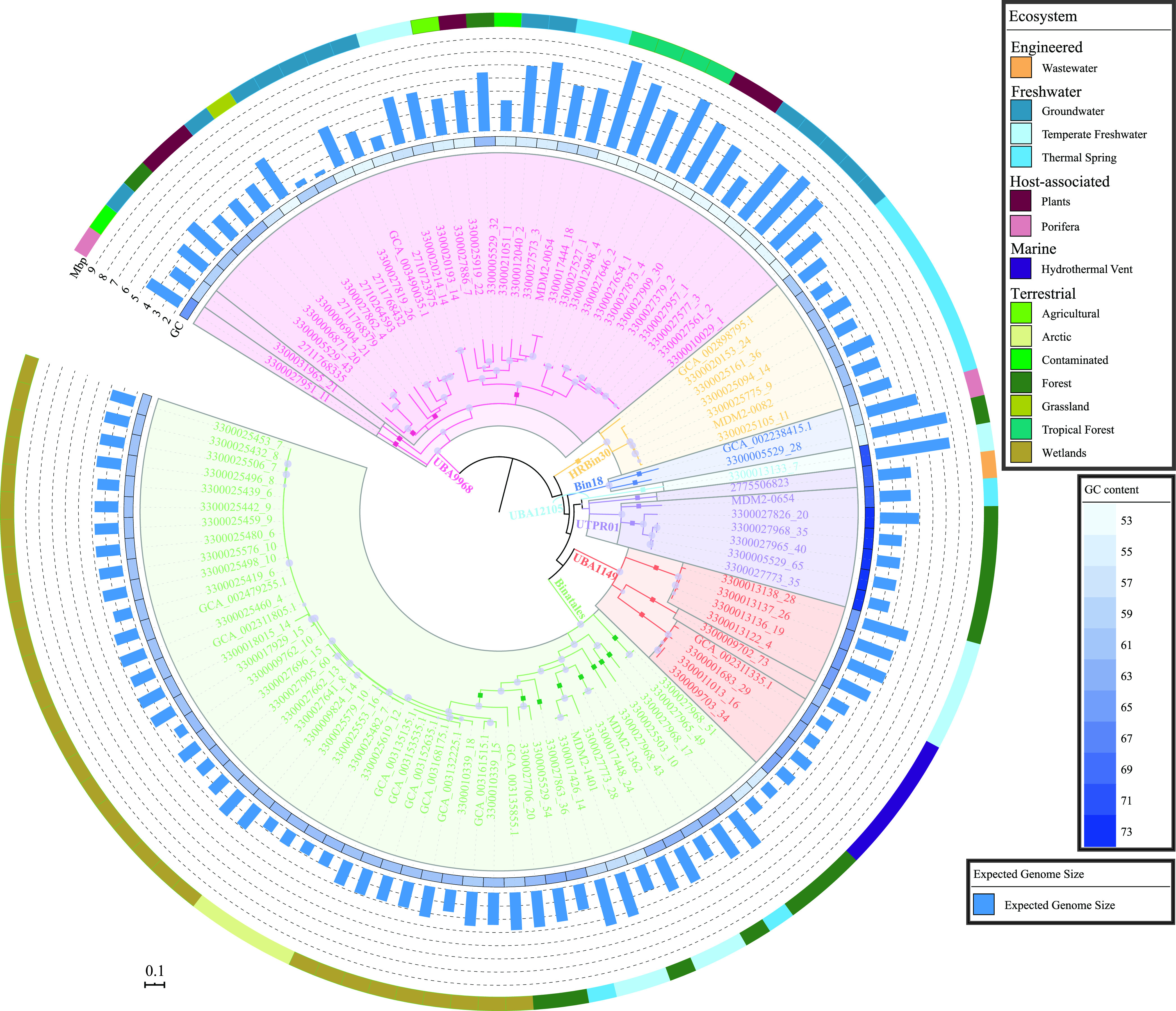
Phylogenomic relationship between analyzed Binatota genomes. The maximum-likelihood tree was constructed in RAxML from a concatenated alignment of 120 single-copy marker genes. The tree was rooted using Deferrisoma camini (GCA_000526155.1) as the outgroup (not shown). Orders are shown as colored wedges: UBA9968, pink; HRBin30, tan; Bin18, blue; UBA12105, cyan; UTPRO1, purple; UBA1149, orange; and Binatales, green. Within each order, families are delineated by gray borders and genera are shown as colored squares on the branches. Bootstrap values are shown as purple bubbles for nodes with ≥70% support. The tracks around the tree represent (innermost-outermost) G+C content (with a heatmap that ranges from 53% [lightest] to 73% [darkest]), expected genome size (bar chart), and classification of the ecosystem from which the genome originated. All genomes analyzed in this study were >70% complete and <10% contaminated. Completion/contamination percentages and individual genomes assembly size are shown in Tables S2 and S3, respectively.

A total of 108 Binatota MAGs with >70% completion and <10% contamination were used for this study, which included 86 medium-quality (>50% completion, <10% contamination) and 22 high-quality (>90% completion, <5% contamination) genomes, as defined by MIMAG standards (15). Binatota genomes clustered into seven orders designated Bin18 (*n* = 2), Binatales (*n* = 48), HRBin30 (*n* = 7), UBA1149 (*n* = 9), UBA9968 (*n* = 34), UBA12105 (*n* = 1), and UTPRO1 (*n* = 7), encompassing 12 families and 24 genera (Fig. 1; Table S1). 16S rRNA gene sequences extracted from orders Bin18 and UBA9968 genomes were classified in SILVA (release 138) (16) as members of class bacteriap25 in the phylum *Myxococcota*, order Binatales, and order HRBin30 as uncultured phylum *RCP2-54* and orders UBA1149 and UTPRO1 as uncultured *Desulfobacterota* classes (Table S1). RDP II-classification (July 2017 release, accessed July 2020) classified all Binatota sequences as unclassified Deltaproteobacteria (Table S1).

10.1128/mBio.00985-21.7 TABLE S1 Binatota genomes used in this study number, their GTDB classification, and the corresponding classification in Silva and RDP databases, the source from which they were obtained, and the calculated Binatota abundances in metagenomes with available contig coverage data. Download Table S1, XLSX file, 0.02 MB. Copyright © 2021 Murphy et al. 2021 Murphy et al. https://creativecommons.org/licenses/by/4.0/ This content is distributed under the terms of the Creative Commons Attribution 4.0 International license.

10.1128/mBio.00985-21.8 TABLE S2 Sequencing statistics for the genomic bins used in this study. Download Table S2, XLSX file, 0.02 MB. Copyright © 2021 Murphy et al. 2021 Murphy et al. https://creativecommons.org/licenses/by/4.0/ This content is distributed under the terms of the Creative Commons Attribution 4.0 International license.

10.1128/mBio.00985-21.9 TABLE S3 General genomic features of the studied genomes. Download Table S3, XLSX file, 0.02 MB. Copyright © 2021 Murphy et al. 2021 Murphy et al. https://creativecommons.org/licenses/by/4.0/ This content is distributed under the terms of the Creative Commons Attribution 4.0 International license.

### Methylotrophy in the Binatota: methanol.

**FIG 2:**
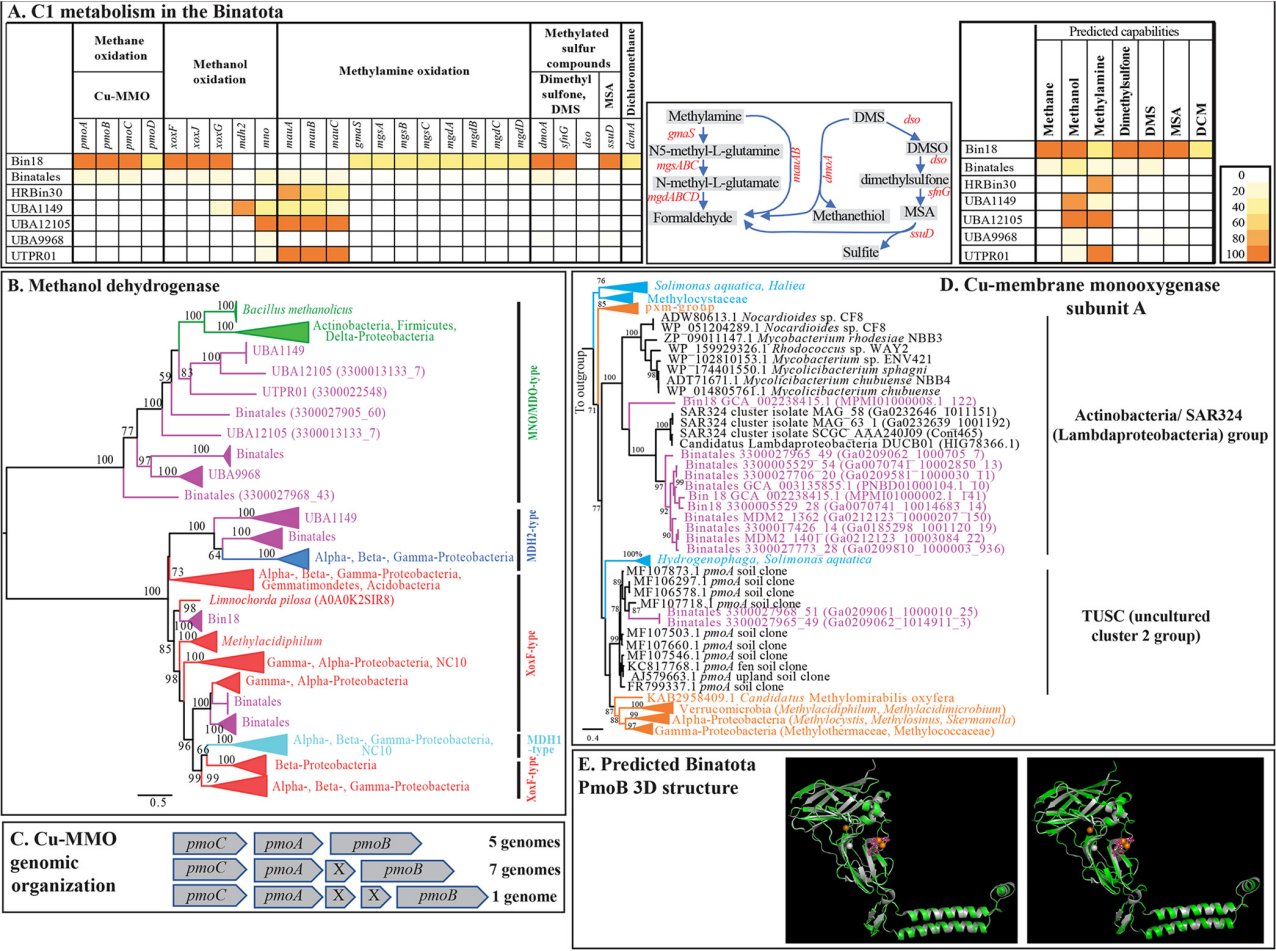
C_1_ substrate degradation capacities in the Binatota. (A) Heatmap of the distribution of various C_1_ oxidation genes in Binatota genomes from different orders. The heatmap colors (as explained in the key) correspond to the percentage of genomes in each order carrying a homologue of the gene in the column header. Pathways involving more than one gene for methylamine and methylated sulfur compounds degradation are shown next to the heatmap. To the right, the per-order predicted C_1_ oxidation capacity is shown as a heatmap with the colors corresponding to the percentage of genomes in each order where the full degradation pathway was detected for the substrate in the column header. These include *pmoABC* for methane, *xoxFJG*, *mdh2*, and/or *mno* for methanol, *mau* and/or indirect glutamate pathway for methylamine, *sfnG* and *ssuD* for dimethylsulfone, *dso*, *sfnG*, and *ssuD* or *dmoA* for dimethylsulfide (DMS), *ssuD* for methane sulfonic acid (MSA), and *dcmA* for dichloromethane (DCM). CuMMO, copper membrane monooxygenase with subunits A, B, C, and D; XoxF-type (*xoxF*, *xoxJ*, *xoxG*), MDH2-type (*mdh2*), and MNO/MDO-type (*mno*) methanol dehydrogenases; direct oxidation methylamine dehydrogenase (*mauABC*); indirect glutamate pathway (*gmaS*, γ-glutamylmethylamide synthase; *mgsABC*, *N*-methyl-l-glutamate synthase; *mgdABCD*, methylglutamate dehydrogenase); dimethylsulfide (DMS) monooxygenase (*dmoA*); dimethyl sulfone monooxygenase (*sfnG*); dimethylsulfide monooxygenase (*dso*); alkane sulfonic acid monooxygenase (*ssuD*); and dichloromethane dehalogenase (*dcmA*). DMSO, dimethyl sulfoxide. (B) Maximum-likelihood phylogenetic tree highlighting the relationship between Binatota methanol dehydrogenases in relation to other methylotrophic taxa. Bootstrap support (from 100 bootstraps) is shown for branches with >50% bootstrap support. (C) Organization of CuMMO genes in Binatota genomes and the number of genomes where each organization was observed. X, hypothetical protein. (D) Maximum-likelihood tree highlighting the relationship between Binatota *pmoA* genes to methanotrophic taxa and environmental amplicons. Bootstrap support (100 bootstraps) is shown for branches with >50% bootstrap support. Sequences from Binatota genomes (shown as order followed by bin name and then PmoA protein ID in parentheses) are in magenta and fall into two clusters: *Actinobacteria*/SAR324 cluster and TUSC uncultured cluster 2. Clusters from previously studied CuMMOs known to reduce methane are in orange, while those known to reduce short-chain alkanes but not methane are in cyan. The tree was rooted using the amoA sequence of “*Candidatus* Nitrosarchaeum limnium SFB1” (EGG41084.1) as an outgroup. (E) Predicted PmoB 3D structure (gray) from a cluster 2 TUSC-affiliated Binatota genome (genome 3300027968_51, left), and an *Actinobacteria*/SAR324-affiliated Binatota genome (genome GCA_002238415.1, right) both superimposed on PmoB from the model methanotroph Methylococcus capsulatus strain Bath (PDB: 3RGB) (green) with global model quality estimation (GMQE) scores of 0.73 and 0.62, respectively.

With the exception of HRBin30, all orders encoded at least one type of methanol dehydrogenase (Figure 2a). Three distinct types of methanol dehydrogenases were identified (Figure 2a and b). (i) The NAD(P)-binding MDO/MNO-type methanol dehydrogenase (*mno*), typically associated with Gram-positive methylotrophic bacteria (*Actinobacteria* and Bacillus methanolicus) (17), was the only type of methanol dehydrogenase identified in orders UBA9968, UBA12105, and UTPRO1 (Figure 2a; Extended Data set 1), as well as some UBA1149 and Binatales genomes. (ii) The MDH2-type methanol dehydrogenase, previously discovered in members of the *Burkholderiales* and *Rhodocyclales* (18), was encountered in the majority of order UBA1149 genomes and in two Binatales genomes. (iii) The lanthanide-dependent pyrroloquinoline quinone (PQQ) methanol dehydrogenase XoxF-type was encountered in nine genomes from the orders Bin18 and Binatales, together with the accessory XoxG c‐type cytochrome and XoxJ periplasmic-binding proteins (Figure 2a). All later genomes also encoded PQQ biosynthesis. Surprisingly, none of the genomes encoded the MxaF1-type (MDH1) methanol dehydrogenase, typically encountered in model methylotrophs (19).

### Methylotrophy in the Binatota: methylamine.

All Binatota orders except UBA9968 encoded methylamine degradation capacity. The direct periplasmic route (methylamine dehydrogenase; *mau*) was more common, with *mauA* and *mauB* enzyme subunits encoded in Binatales, HRBin30, UBA1149, UBA12105, and UTPRO1 (Figure 2a; Extended Data set 1). Amicyanin (encoded by *mauC*) is the most probable electron acceptor for methylamine dehydrogenase (19) (Figure 2a). On the other hand, one Bin18 genome and two Binatales genomes (that also encode the *mau* cluster) carried the full complement of genes for methylamine oxidation via the indirect glutamate pathway (Figure 2a; Extended Data set 1).

### Methylotrophy in the Binatota: methylated sulfur compounds.

Binatota genomes encoded several enzymes involved in the degradation of dimethyl sulfone, methane sulfonic acid (MSA), and dimethyl sulfide (DMS). Nine genomes (2 Bin18 and 7 Binatales) encoded dimethyl sulfone monooxygenase (*sfnG*) involved in the degradation of dimethyl sulfone to MSA with the concomitant release of formaldehyde. Three of these nine genomes also encoded alkane sulfonic acid monooxygenase (*ssuD*), which will further degrade the MSA to formaldehyde and sulfite. Degradation of DMS via DMS monooxygenase (*dmoA*) to formaldehyde and sulfide was encountered in 13 genomes (2 Bin18, 9 Binatales, and 2 UBA9968). Further, one Binatales genome encoded the *dso* system (enzyme class [EC]: 1.14.13.245) for DMS oxidation to dimethyl sulfone, which could be further degraded to MSA as explained above (Figure 2a; Extended Data set 1).

### Methylotrophy in the Binatota: dihalogenated methane.

One Bin18 genome encoded the specific dehalogenase/glutathione *S*-transferase (*dcmA*) capable of converting dichloromethane to formaldehyde.

### Methylotrophy in the Binatota: methane.

Genes encoding copper membrane monooxygenases (CuMMOs), a family of enzymes that includes particulate methane monooxygenase (pMMO), were identified in orders Bin18 (2/2 genomes) and Binatales (9/48 genomes) (Figure 2a; Extended Data set 1), while genes encoding soluble methane monooxygenase (sMMO) were not found. A single copy of the three genes encoding all CuMMO subunits (A, B, and C) was encountered in 9 of the 11 genomes, while two copies were identified in two genomes. CuMMO subunit-encoding genes (A, B, and C) occurred as a contiguous unit in all genomes, with a CAB (5 genomes) and/or CAxB or CAxxB (8 genomes, where x is a hypothetical protein) organization, similar to the pMMO operon structure in methanotrophic *Proteobacteria*, *Verrucomicrobia*, and “*Candidatus* Methylomirabilis” (NC10) (20–23) (Figure 2c). In addition, 5 of the above-mentioned 11 genomes also encoded a *pmoD* subunit, recently suggested to be involved in facilitating the enzyme complex assembly and/or in electron transfer to the enzyme’s active site (24, 25). Phylogenetic analysis of Binatota *pmoA* sequences revealed their affiliation with two distinct clades: the yet-uncultured cluster 2 TUSC (tropical upland soil cluster) methanotrophs (26) (2 Binatales genomes) and a clade encompassing *bmoA* sequences (putative butane monooxygenase gene A) from *Actinobacteria* (*Nocardioides* sp. strain CF8, *Mycolicibacterium*, and *Rhodococcus*) and SAR324 (“*Candidatus* Lambdaproteobacteria”) (27, 28) (Figure 2d). Members harboring these specific lineages have previously been identified in a wide range of environments, including soil (26). Previous studies have linked cluster 2 TUSC CuMMO-harboring organisms to methane oxidation based on selective enrichment on methane in microcosms derived from Lake Washington sediments (29). Binatota genomes encoding TUSC-affiliated CuMMO also harbored genes for downstream methanol and formaldehyde oxidation as well as formaldehyde assimilation (see below), providing further evidence for their putative involvement in methane oxidation. On the other hand, studies on *Nocardioides* sp. strain CF8 demonstrated its capacity to oxidize short-chain (C_2_ to C_4_) hydrocarbons, but not methane, via its CuMMO, and its genome lacked methanol dehydrogenase homologues (30). Such data favor a putative short-chain hydrocarbon degradation function for organisms encoding this type of CuMMO, although we note that five out of the nine Binatota genomes carrying SAR324/*Actinobacteria*-affiliated *pmoA* sequences also encoded at least one methanol dehydrogenase homologue. Modeling CuMMO subunits from both TUSC-type and *Actinobacteria*/SAR324-type Binatota genomes using Methylococcus capsulatus (Bath) 3D model (Protein Data Bank ID: 3RGB) revealed a heterotrimeric structure (α_3_β_3_γ_3_) with the 7, 2, and 5 alpha helices of the PmoA, PmoB, and PmoC subunits, respectively, as well as the beta sheets characteristic of PmoA and PmoB subunits (Fig. S1). Recently, the location of the active site at the amino terminus of the PmoB subunit has been suggested (31). There has been recent debate as to the exact nuclearity of the Cu cofactor at the active site (31–33). Regardless of the nuclearity of the copper metal center, conserved histidine residues His^33^, His^137^, and His^139^ (numbering following the Methylococcus capsulatus strain Bath PmoB subunit, Protein DataBank ID: 3RGB), thought to coordinate the Cu cofactor, were identified in all TUSC-affiliated and SAR324/*Actinobacteria*-affiliated Binatota CuMMO sequences (Fig. S1). Modeling PmoB subunits from both TUSC-type and *Actinobacteria*/SAR324-type Binatota genomes using Methylococcus capsulatus (Bath) PmoB subunit (Protein Data Bank [PDB] ID: 3RGB) predicted the binding pockets for Cu in Binatota sequences (Figure 2e).

10.1128/mBio.00985-21.2 FIG S1 (A) Alignment of the PmoB subunit of the 11 copper membrane monooxygenases predicted in Binatota genomes to PmoB from Methylococcus capsulatus (PDB ID: 3RGB). The alignment is showing conserved residues (red highlight). Of particular importance are the three conserved histidine residues His33, His137, and His139 (shown with a blue rectangle), thought to coordinate the Cu cofactor. Numbering follows the Methylococcus capsulatus strain Bath PmoB subunit (PDB: 3RGB). Alignment was created using the ENDscript webserver (http://espript.ibcp.fr/ESPript/ESPript/). (B and C) Predicted Cu methane monooxygenase (PmoABC) 3D structure (grey) from a cluster 2 TUSC-affiliated Binatota genome (genome 3300027968_51; panel B) and an *Actinobacteria*/SAR324-affiliated Binatota genome (genome GCA_002238415.1; panel C), both superimposed on CuMMO from the model methanotroph Methylococcus capsulatus strain Bath (PDB: 3RGB) (green) with a global model quality estimate of 0.7 and 0.62, respectively, and a quaternary structure quality score of 0.57 and 0.55, respectively. Download FIG S1, PDF file, 1.4 MB. Copyright © 2021 Murphy et al. 2021 Murphy et al. https://creativecommons.org/licenses/by/4.0/ This content is distributed under the terms of the Creative Commons Attribution 4.0 International license.

As previously noted (19), methylotrophy requires the possession of three metabolic modules: C_1_ oxidation to formaldehyde, formaldehyde oxidation to CO_2_, and formaldehyde assimilation. Formaldehyde generated by C_1_ substrates oxidation is subsequently oxidized to formate and eventually CO_2_. Multiple pathways for formaldehyde oxidation to formate were identified in all Binatota orders (Text S1; Fig. S2). In addition, the majority of Binatota genomes encoded a formate dehydrogenase for formate oxidation to CO_2_ (Text S1; Fig. S2). Finally, for assimilating formaldehyde into biomass, genes encoding all enzymes of the serine cycle, as well as genes encoding different routes of glyoxylate regeneration, were identified in all genomes (Text S1; Fig. S2).

10.1128/mBio.00985-21.1 TEXT S1 Details of the Binatota metabolic capabilities discussed in the main text are shown in the supplementary text file, including formaldehyde oxidation to CO_2_, formaldehyde assimilation, alkane degradation, and predicted electron transport chain. Download Text S1, PDF file, 0.3 MB. Copyright © 2021 Murphy et al. 2021 Murphy et al. https://creativecommons.org/licenses/by/4.0/ This content is distributed under the terms of the Creative Commons Attribution 4.0 International license.

10.1128/mBio.00985-21.3 FIG S2 Formaldehyde oxidation and assimilation capabilities encoded by Binatota genomes. (A) Heatmap of the distribution of formaldehyde oxidation genes in Binatota genomes from different orders. The heatmap colors (as explained in the key) correspond to the percentage of genomes in each order encoding a homologue of the gene in the column header. Shown are the different routes of formaldehyde oxidation, including the (myco)thiol-dependent formaldehyde dehydrogenase *fadH/mscR* (along with mycothiol biosynthesis genes [*mshABC*]), the H_4_F-linked pathway (comprising the genes bifunctional methylene-H_4_F dehydrogenase and methenyl-H_4_F cyclohydrolase [*folD*], reversible formyl-H_4_F ligase [*ftfL*], and irreversible formyl-H_4_F hydrolase [*purU*]), the glutathione-independent formaldehyde dehydrogenase (*fdhA*), and the glutathione-dependent formaldehyde [comprising the S-(hydroxymethyl)glutathione synthase (*gfa*), NAD- and glutathione-dependent formaldehyde dehydrogenase (*frmA*), and S-formylglutathione hydrolase (*frmB*)]. Also shown is the distribution of the NAD-dependent formate dehydrogenase (EC: 1.17.1.9) (*fdh*) for formate oxidation. (B) Overview of the pathways for formaldehyde assimilation via the serine cycle (left) and glyoxylate regeneration via the ethylmalonyl-CoA pathway and the glyoxylate shunt (GS) (right). Names of enzymes are shown in red and their distribution in the Binatota genomes from different orders is shown in the heatmap in panel C. *glyA*, glycine hydroxymethyltransferase [EC: 2.1.2.1]; *sgaA*, serine-glyoxylate transaminase [EC: 2.6.1.45]; *hprA*, glycerate dehydrogenase [EC: 1.1.1.29]; *gck*, glycerate 2-kinase [EC: 2.7.1.165]; *ppc*, phosphoenolpyruvate carboxylase [EC: 4.1.1.31]; *pckA*, phosphoenolpyruvate carboxykinase; *mdh*, malate dehydrogenase [EC: 1.1.1.37]; *mtkA/B*, malate-CoA ligase [EC: 6.2.1.9]; *mcl*, malyl-CoA/(S)-citramalyl-CoA lyase [EC: 4.1.3.24 4.1.3.25]; *aceA*, isocitrate lyase [EC: 4.1.3.1]; *aceB*, malate synthase [EC: 2.3.3.9]; *phbB*, acetoacetyl-CoA reductase [EC: 1.1.1.36]; *croR*, 3-hydroxybutyryl-CoA dehydratase [EC: 4.2.1.55]; *ccr*, crotonyl-CoA carboxylase/reductase [EC: 1.3.1.85]; *epi*, methylmalonyl-CoA/ethylmalonyl-CoA epimerase [EC: 5.1.99.1]; *ecm*, ethylmalonyl-CoA mutase [EC: 5.4.99.63]; *mcd*, (2S)-methylsuccinyl-CoA dehydrogenase [EC: 1.3.8.12]; *mch*, 2-methylfumaryl-CoA hydratase [EC: 4.2.1.148]; *mut*, methylmalonyl-CoA mutase [EC: 5.4.99.2]; *mcmA1/A2*, methylmalonyl-CoA mutase [EC: 5.4.99.2]. Abbreviations: PEP, phosphoenol pyruvate; OAA, oxaloacetate. Download FIG S2, PDF file, 0.2 MB. Copyright © 2021 Murphy et al. 2021 Murphy et al. https://creativecommons.org/licenses/by/4.0/ This content is distributed under the terms of the Creative Commons Attribution 4.0 International license.

### Alkane degradation in the Binatota.

**FIG 3:**
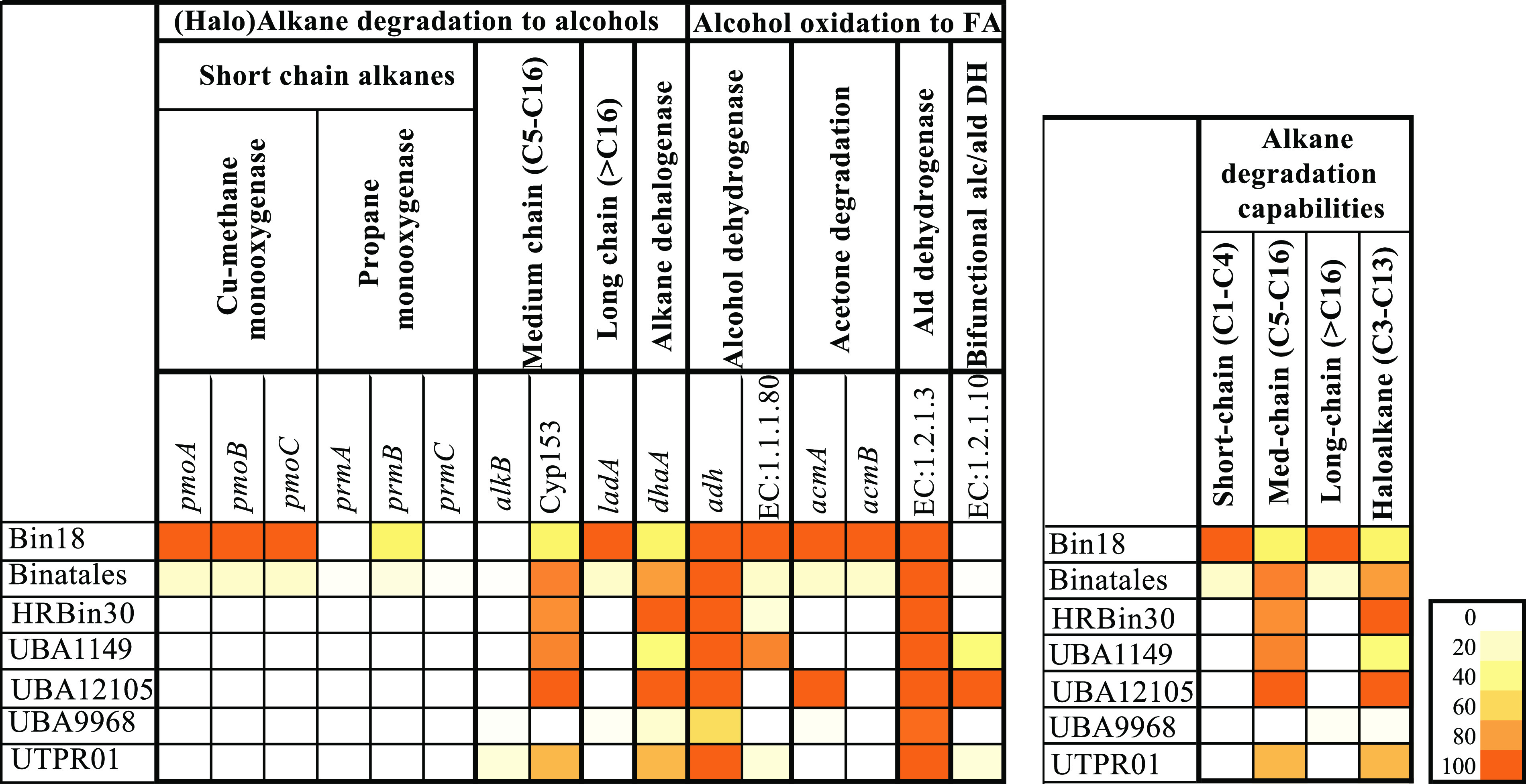
Heatmap of the distribution of (halo)alkane degradation to alcohol in Binatota genomes. The heatmap colors (as explained in the key) correspond to the percentage of genomes in each order carrying a homologue of the gene in the column header. The per-order predicted alkane-degradation capacity is shown to the right as a heatmap with the colors corresponding to the percentage of genomes in each order where the full degradation pathway was detected for the substrate in the column header. These include CuMMO and/or *prmABC* for short-chain alkanes, *alkB* or Cyp153 for medium-chain alkanes, *ladA* for long-chain alkanes, and *dhaA* for haloalkanes. Ald, aldehyde.

In addition to methylotrophy and methanotrophy, Binatota genomes exhibited extensive short-, medium-, and long-chain alkanes degradation capabilities. In addition to the putative capacity of *Actinobacteria*/SAR324-affiliated CuMMO to oxidize C_1_ to C_5_ alkanes and C_1_ to C_4_ alkenes as described above, some Binatota genomes encoded propane-2-monoxygenase (*prmABC*), an enzyme mediating propane hydroxylation in the 2-position yielding isopropanol. Several genomes also encoded medium-chain-specific alkane hydroxylases, e.g., homologues of the nonheme iron *alkB* (34) and Cyp153-class alkane hydroxylases (35). The genomes also encoded multiple long-chain specific alkane monooxygenases, e.g., *ladA* homologues (enzyme class [EC]: 1.14.14.28) (36) (Fig. 3, Extended Data set 1). Finally, Binatota genomes encoded the capacity to metabolize medium-chain haloalkane substrates. All genomes encoded *dhaA* (haloalkane dehalogenases [EC: 3.8.1.5]) known to have a broad substrate specificity for medium-chain-length (C_3_ to C_10_) mono- and dihaloalkanes, resulting in the production of their corresponding primary alcohol and haloalcohols, respectively (37) (Fig. 3, Extended Data set 1).

Alcohol and aldehyde dehydrogenases sequentially oxidize the resulting alcohols to their corresponding fatty acids or fatty acyl-CoA. Binatota genomes encode a plethora of alcohol and aldehyde dehydrogenases mediating such processes (Text S1; Fig. S3). As well, a complete fatty acid degradation machinery that enables all orders of the Binatota to degrade short-, medium-, and long-chain fatty acids to acetyl-CoA and propionyl-CoA was identified (Text S1; Fig. S3).

10.1128/mBio.00985-21.4 FIG S3 (A) Heatmap of the distribution of various chain-length fatty acid and haloacid degradation genes in Binatota genomes. The heatmap colors (as explained in the key) correspond to the percentage of genomes in each order encoding a homologue of the gene in the column header. (B) Propionyl-CoA degradation pathways encoded by the Binatota genomes. The methylmalonyl-CoA (MMCoA) pathway is shown in blue, while the 2-methylcitrate pathway is shown in green. In some genomes, the MMCoA pathway seems to be functional but with a slight modification (shown in purple) that includes glyoxylate assimilation and regeneration. *pmoABC*, copper membrane monooxygenase with denoting subunits A, B, and C; *prmABC*, propane 2-monooxygenase [EC: 1.14.13.227]; *alkB*, alkane 1-monooxygenase [EC: 1.14.15.3]; cyp153, cytochrome P450 alkane hydroxylase [EC 1.14.15.1]; *ladA*, long-chain alkane monooxygenase [EC: 1.14.14.28]; *dhaA*, haloalkane dehalogenase [EC: 3.8.1.5]; *adh*, alcohol dehydrogenase [EC: 1.1.1.1]; isopropanol dehydrogenase (NADP+) [EC: 1.1.1.80]; *acmA*, acetone monooxygenase (methyl acetate-forming) [EC: 1.14.13.226]; *acmB*, methyl acetate hydrolase [EC: 3.1.1.114]; aldehyde dehydrogenase (NAD+) [EC: 1.2.1.3]; acetaldehyde dehydrogenase (acetylating) [EC: 1.2.1.10]; *acdAB*, acetate-CoA ligase (ADP-forming) [EC: 6.2.1.13]; acs, acetyl-CoA synthase [EC: 2.3.1.169]; *atoAD*, acetate-CoA/acetoacetate CoA-transferase [EC: 2.8.3.8 2.8.3.9]; medium-chain acyl-CoA synthetase [EC: 6.2.1.2]; *fadD*, long-chain acyl-CoA synthetase [EC: 6.2.1.3]; *pccA*, propionyl-CoA carboxylase alpha chain [EC: 6.4.1.3]; *epi*, methylmalonyl-CoA/ethylmalonyl-CoA epimerase [EC: 5.1.99.1]; *mut*, methylmalonyl-CoA mutase [EC: 5.4.99.2]; *mcl*, malyl-CoA/(S)-citramalyl-CoA lyase [EC: 4.1.3.24 4.1.3.25]; *mch*, 2-methylfumaryl-CoA hydratase [EC: 4.2.1.148]; *mct*, 2-methylfumaryl-CoA isomerase [EC: 5.4.1.3]; *meh*, 3-methylfumaryl-CoA hydratase [EC: 4.2.1.153]; *smtAB*, succinyl-CoA:(S)-malate-CoA-transferase subunit A [EC: 2.8.3.22]; *prpB*, methylisocitrate lyase [EC: 4.1.3.30]; *prpC*, 2-methylcitrate synthase [EC: 2.3.3.5]; *prpD*, 2-methylcitrate dehydratase [EC: 4.2.1.79]; *bcd*, butyryl-CoA dehydrogenase [EC: 1.3.8.1]; *acd*, acyl-CoA dehydrogenase [EC: 1.3.8.7]; *paaF*, enoyl-CoA hydratase [EC: 4.2.1.17]; *crt*, enoyl-CoA hydratase [EC: 4.2.1.17]; *paaH*, 3-hydroxybutyryl-CoA dehydrogenase [EC: 1.1.1.157]; *phbB*, acetoacetyl-CoA reductase [EC: 1.1.1.36]; *atoB*, acetyl-CoA C-acetyltransferase [EC: 2.3.1.9]; *fadJ*, 3-hydroxyacyl-CoA dehydrogenase/enoyl-CoA hydratase/3-hydroxybutyryl-CoA epimerase [EC: 1.1.1.35 4.2.1.17 5.1.2.3]; *fadA*, acetyl-CoA acyltransferase [EC: 2.3.1.16]; *dehH*, 2-haloacid dehalogenase [EC: 3.8.1.2]; haloacetate dehalogenase [EC: 3.8.1.3]; *glcDEF*, glycolate oxidase [EC: 1.1.3.15]; (S)-2-hydroxy-acid oxidase [EC: 1.1.3.15]. Download FIG S3, PDF file, 0.3 MB. Copyright © 2021 Murphy et al. 2021 Murphy et al. https://creativecommons.org/licenses/by/4.0/ This content is distributed under the terms of the Creative Commons Attribution 4.0 International license.

### Predicted electron transport chain.

**FIG 4:**
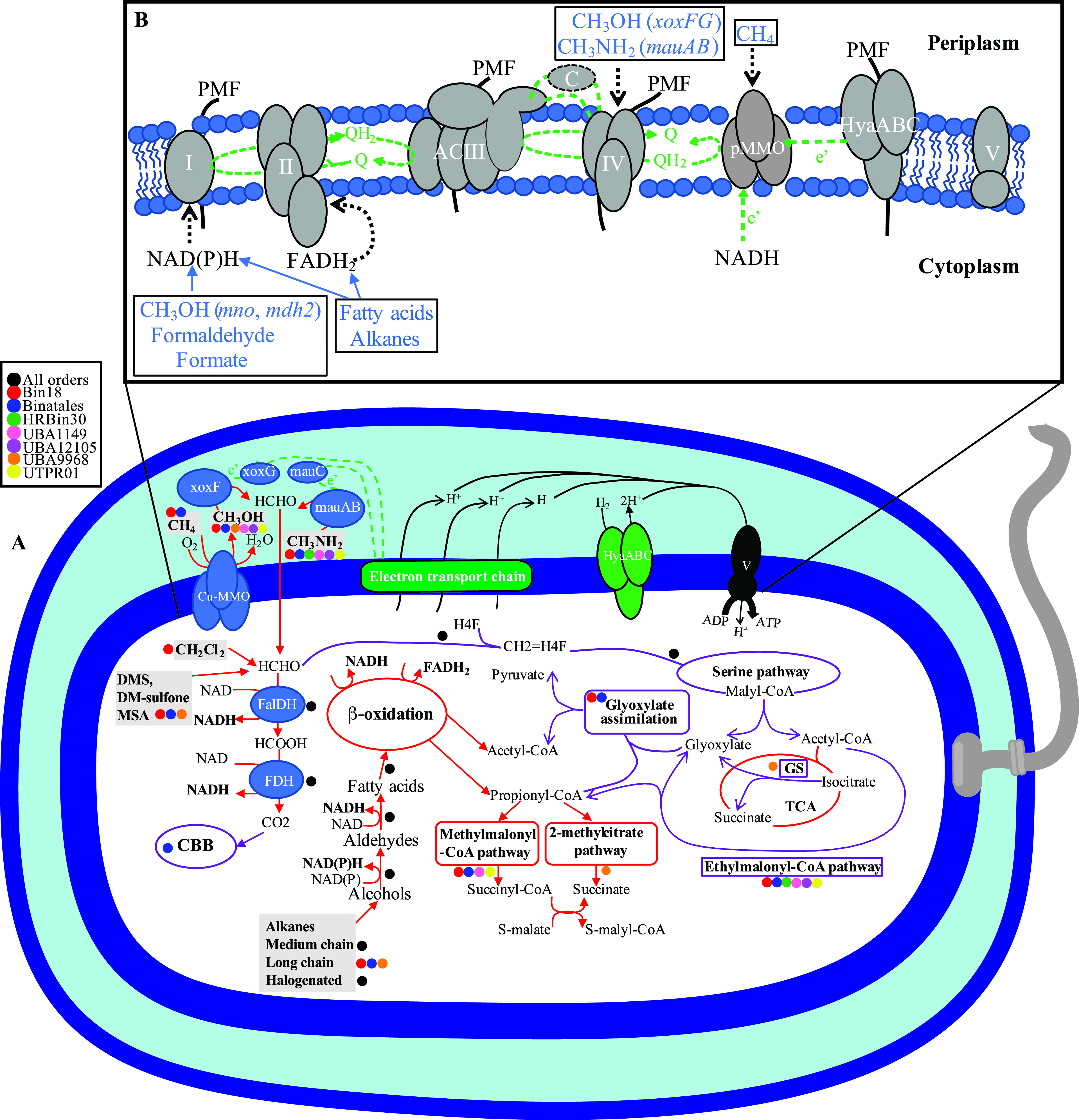
(A) Cartoon depicting different metabolic capabilities encoded in the Binatota genomes with capabilities predicted for different orders shown as colored circles as shown in the legend. Enzymes for C_1_ metabolism are shown in blue and include the copper membrane monooxygenases (CuMMOs), methanol dehydrogenase (*xoxFG*), and methylamine dehydrogenase (*mauABC*), as well as the cytoplasmic formaldehyde dehydrogenase (FalDH) and formate dehydrogenase (FDH). Electron transport chain is shown as a green rectangle. Electron transfer from periplasmic enzymes to the ETC is shown as dotted green lines. The sites of proton extrusion to the periplasm are shown as black arrows, as is the F-type ATP synthase. Carbon dissimilation routes are shown as red arrows, while assimilatory routes are shown as purple arrows. Details of the assimilatory pathways are shown in Fig. S2 and S3. Reducing equivalents potentially fueling the ETC [NAD(P)H and FADH_2_] are shown in boldface. All substrates predicted to support growth are shown in boldface within gray boxes. A flagellum is depicted, the biosynthetic genes of which were identified in genomes belonging to all orders except Bin18, HRBin30, and UBA1149. The cell is depicted as rod-shaped based on the identification of the rod shape determining gene *rodA* in all genomes and the rod-shape determining genes *mreB* and *mreC* in genomes from all orders except UBA1149. The inset on top (B) details the electron transport chain in the Binatota with all electron transfer complexes (I, II, ACIII, IV) embedded in the inner membrane, along with the particulate methane monooxygenase (pMMO) and the H_2_-uptake [NiFe] hydrogenase (HyaABC). All genomes also encoded an F-type ATP synthase complex (V). Substrates potentially supporting growth are shown in blue with predicted entry points to the ETC shown as dotted black arrows. Sites of proton extrusion to the periplasm and proton motive force (PMF) creation are shown as solid black lines, while sites of electron (e’) transfer are shown as dotted green lines. Three possible physiological reductants are shown for pMMO (as dotted green arrows): the quinone pool coupled to ACIII, NADH, and/or some of the reduced quinones generated through H_2_ oxidation by HyaABC. Abbreviations: CBB, Calvin Benson Bassham cycle; FalDH, NAD-linked glutathione-independent formaldehyde dehydrogenase, *fdhA*; FDH, NAD-dependent formate dehydrogenase (EC: 1.17.1.9); Fum, fumarate; GS, glyoxylate shunt; H_4_F, tetrahydrofolate; HyaABC, type I respiratory O_2_-tolerant H_2_-uptake [NiFe] hydrogenase; *mauABC*, methylamine dehydrogenase; CuMMO. copper membrane monooxygenases; *xoxFG*, xoxF-type methanol dehydrogenase; succ, succinate; TCA, tricarboxylic acid cycle; V, F-type ATP synthase (EC: 7.1.2.2 7.2.2.1).

All Binatota genomes encode an aerobic respiratory chain comprising complexes I and II, alternate complex III (ACIII, encoded by *actABCDEFG*), and complex IV, as well as an F-type H^+^-translocating ATP synthase (Text S1; Fig. 4). Binatota genomes also encode respiratory O_2_-tolerant H_2_-uptake [NiFe] hydrogenases, belonging to groups 1c (6 sequences), 1f (22 sequences), 1i (1 sequence), and 1h (4 sequences) (Fig. S4). Simultaneous oxidation of hydrogen (via type I respiratory O_2_-tolerant hydrogenases) and methane (via pMMO) has been shown to occur in methanotrophic *Verrucomicrobia* to maximize proton-motive force generation and subsequent ATP production (38). As well, some of the reduced quinones generated through H_2_ oxidation are thought to provide reducing power for catalysis by pMMO (38) (Fig. 4). Details on the distribution of electron transport chain (ETC) components across Binatota orders are shown in Fig. S4, and the proposed electron flow under different growth conditions is presented in Text S1.

10.1128/mBio.00985-21.5 FIG S4 Electron transport chain in the Binatota. (A) Heatmap of the distribution of electron transport chain components in the Binatota genomes and electrons entry points from various substrates. The heatmap colors (as explained in the key) correspond to the percentage of genomes in each order carrying a homologue of the gene in the column header. All subunits of complexes I (NADH-quinone oxidoreductase [EC: 7.1.1.2]) and II (succinate dehydrogenase/fumarate reductase [EC: 1.3.5.1 1.3.5.4]) were encoded in all genomes but are shown here as single components for ease of visualization. Genes encoding quinone-cytochrome C reductase activities belonged to complex III (cytochrome bc1; ISP/*cytb*/cyt1) and/or alternate cytochrome III (ACIII; *actABCDEF*), while genes encoding cytochrome c oxidase activities (complex IV) belonged to different families, including family A (cytochrome *c* oxidase aa3; *coxABC*), family C (cytochrome *c* oxidase cbb3; *ccoNOP*), and/or cytochrome *bd* (*cydAB*). Possible electron transfer proteins between complex III (or alternate complex III) and complex IV belonging to different cytochrome *c* families are shown. Also shown in panel A is the distribution of the three subunits of the type I respiratory O_2_-tolerant H_2_-uptake [NiFe] hydrogenase (*hyaABC*) in Binatota genomes. (B) Maximum-likelihood phylogenetic tree showing the classification of the *hyaA* genes carried by the Binatota genomes (magenta) in relation to other [NiFe] hydrogenases. The [Fe-Fe] hydrogenase of Methanobacterium formicicum was used as the outgroup. Bootstrap support (from 100 bootstraps) is shown for branches with >50% support. Download FIG S4, PDF file, 0.3 MB. Copyright © 2021 Murphy et al. 2021 Murphy et al. https://creativecommons.org/licenses/by/4.0/ This content is distributed under the terms of the Creative Commons Attribution 4.0 International license.

### Pigment production genes in the Binatota.

**FIG 5:**
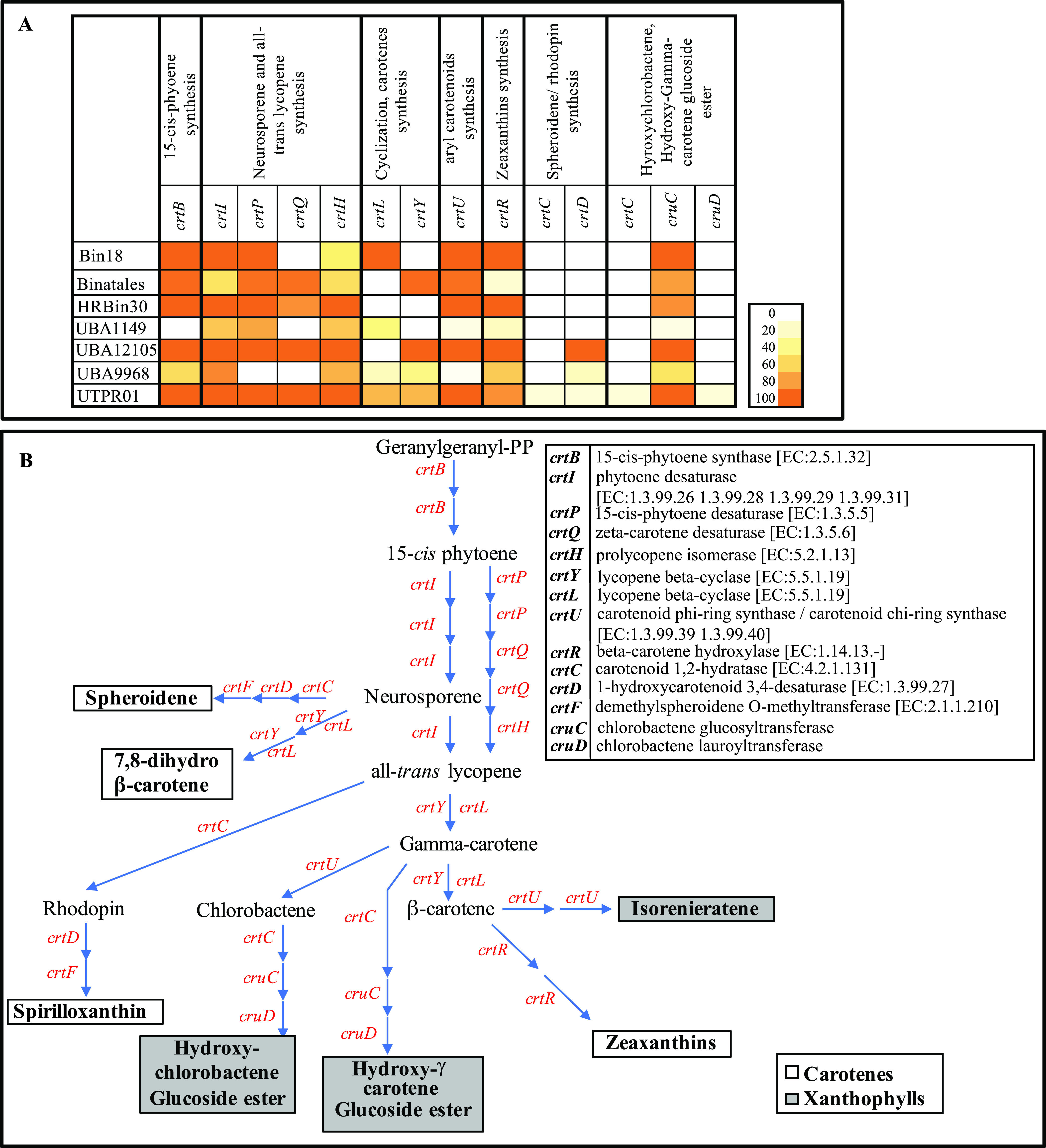
Carotenoids biosynthesis capabilities in Binatota genomes. (A) Distribution of carotenoid biosynthesis genes in the Binatota genomes. The heatmap colors (as explained in the key) correspond to the percentage of genomes in each order encoding a homologue of the gene in the column header. (B) Carotenoid biosynthesis scheme in Binatota based on the identified genes. Genes encoding enzymes catalyzing each step are shown in red and their descriptions with EC numbers are shown to the right. Binatota genomes encode the capability to biosynthesize both exclusively hydrocarbon carotenes (white boxes) or the oxygenated xanthophylls (gray boxes).

**Carotenoids.** Analysis of the Binatota genomes demonstrated a wide range of hydrocarbon (carotenes) and oxygenated (xanthophyll) carotenoid biosynthesis capabilities. Carotenoids biosynthetic machinery in the Binatota included *crtB* for 15-cis-phyotene synthesis from geranylgeranyl pyrophosphate (PP), *crtI*, *crtP*, *crtQ*, and *crtH* for neurosporene and all-*trans* lycopene formation from 15-cis-phytone, *crtY* or *crtL* for gamma- and beta-carotene formation from all-*trans* lycopene, and a wide range of genes encoding enzymes for the conversion of neurosporene to spheroidene and 7,8-dihydro β-carotene, as well as the conversion of all-*trans* lycopene to spirilloxanthin, gamma-carotene to hydroxy-chlorobactene glucoside ester and hydroxy-γ-carotene glucoside ester, and beta-carotene to isorenieratene and zeaxanthins (Fig. 5a and b; Extended Data set 1). Gene distribution pattern (Figure 5a; Extended Data set 1) predicts that all Binatota orders are capable of neurosporene and all-*trans* lycopene biosynthesis, and all but the order HRBin30 are capable of isorenieratene, zeaxanthin, β-carotene, and dihydro β-carotene biosynthesis and with specialization of order UTPRO1 in spirilloxanthin, spheroidene, hydroxy-chlorobactene, and hydroxy-γ-carotene biosynthesis.

**FIG 6:**
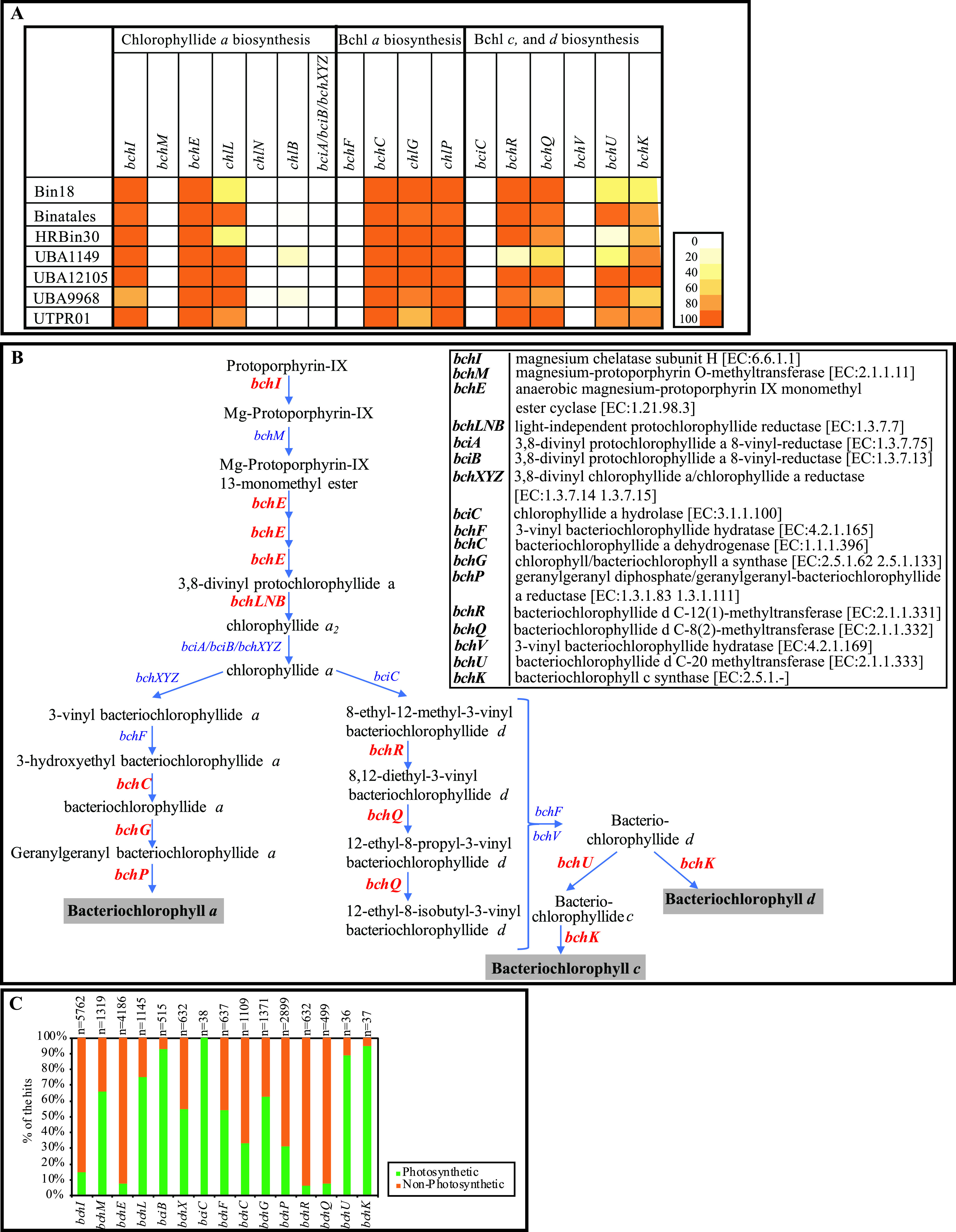
Bacteriochlorophylls biosynthesis genes encountered in Binatota genomes studied suggesting an incomplete pathway for bacteriochlorophyll *a*, *c*, and/or *d* biosynthesis. (A) Distribution of chlorophyll biosynthesis genes in Binatota genomes. The heatmap colors (as explained in the key) correspond to the percentage of genomes in each order carrying a homologue of the gene in the column header. (B) Bacteriochlorophylls biosynthesis pathway. Genes identified in at least one Binatota genome are shown in red boldface text, while these with no homologues in the Binatota genomes are shown in blue text. Gene descriptions with EC numbers are shown to the right of the figure. (C) Distribution patterns of bacteriochlorophyll biosynthesis genes. The search was conducted in the functionally annotated bacterial tree of life AnnoTree (75) using single KEGG orthologies implicated in chlorophyll biosynthesis. Gene names are shown on the *x* axis, total number of hits is shown above the bar for each gene, and the percentage of hits in genomes from photosynthetic (green) versus nonphotosynthetic (orange) genera are in the stacked bars.

**Bacteriochlorophylls.** Surprisingly, homologues of multiple genes involved in bacteriochlorophyll biosynthesis were ubiquitous in Binatota genomes (Figure 6a to c). Bacteriochlorophyll biosynthesis starts with the formation of chlorophyllide *a* from protoporphyrin IX (Figure 6b). Within this pathway, genes encoding the first *bchI* (Mg-chelatase [EC: 6.6.1.1]), third *bchE* (magnesium-protoporphyrin IX monomethyl ester cyclase [EC: 1.21.98.3]), and fourth *bchLNB* (3,8-divinyl protochlorophyllide reductase [EC: 1.3.7.7]) steps were identified in the Binatota genomes (Fig. 6a and b; Extended Data set 1). However, homologues of genes encoding the second *bchM* (magnesium-protoporphyrin O-methyltransferase [EC: 2.1.1.11]) and the fifth *bciA* or *bicB* (3,8-divinyl protochlorophyllide *a* 8-vinyl-reductase) or *bchXYZ* (chlorophyllide *a* reductase [EC 1.3.7.15]) steps were absent (Figure 6a and b). A similar patchy distribution was observed in the pathway for bacteriochlorophyll *a* (BChl *a*) formation from chlorophyllide *a* (Figure 6b), where genes encoding *bchXYZ* (chlorophyllide *a* reductase [EC 1.3.7.15]) and *bchF* (chlorophyllide *a* 3^1^-hydratase [EC 4.2.1.165]) were not identified, while genes encoding *bchC* (bacteriochlorophyllide *a* dehydrogenase [EC 1.1.1.396]), *bchG* (bacteriochlorophyll *a* synthase [EC: EC: 2.5.1.133]), and *bchP* (geranylgeranyl-bacteriochlorophyllide *a* reductase [EC 1.3.1.111]) were present in most genomes (Figure 6a; Extended Data set 1). Finally, within the pathway for bacteriochlorophylls *c* (BChl *c*) and *d* (BChl *d*) formation from chlorophyllide *a* (Figure 6b), genes for *bciC* (chlorophyllide *a* hydrolase [EC: 3.1.1.100]) and *bchF* (chlorophyllide *a* 3^1^-hydratase [EC: 4.2.1.165]) or *bchV* (3-vinyl bacteriochlorophyllide hydratase [EC: 4.2.1.169]) were not identified, while genes for *bchR* [bacteriochlorophyllide *d* C-12(1)-methyltransferase (EC: 2.1.1.331)], *bchQ* [bacteriochlorophyllide *d* C-8(2)-methyltransferase (EC: 2.1.1.332)], *bchU* (bacteriochlorophyllide *d* C-20 methyltransferase [EC: 2.1.1.333]), and *bchK* (bacteriochlorophyll *c* synthase [EC: 2.5.1.-]) were identified (Figure 6b; Extended Data set 1).

### Ecological distribution of the Binatota.

**FIG 7:**
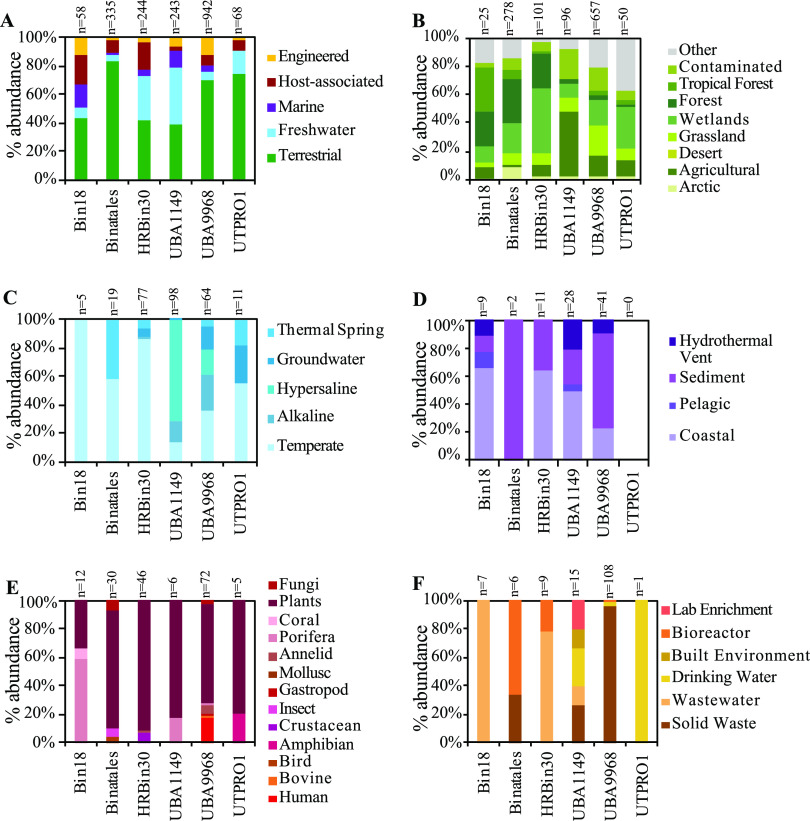
Ecological distribution of Binatota-affiliated 16S rRNA sequences in GenBank nt database. Binatota orders are shown on the *x* axis, while percent abundance in different environments (classified based on the GOLD ecosystem classification scheme) is shown on the *y* axis (A). Further subclassifications for each environment are shown for (B) terrestrial, (C) freshwater, (D) marine, (E) host-associated, and (F) engineered environments. The total number of hit sequences for each order is shown above the bar graphs. Details, including GenBank accession number of hit sequences, are shown in Extended Data 2. Order UBA12105 genome assembly did not contain a16S rRNA gene, so this order is not included in the analysis.

A total of 1,889 (GenBank nucleotide database [GenBank nt]) and 1,213 (IMG/M) 16S rRNA genes affiliated with the Binatota orders were identified (Extended Data set 2; Fig. 7; Fig. S5a). Analyzing their environmental distribution showed preference of Binatota to terrestrial soil habitats (39.5 to 83.0% of GenBank, 31.7 to 91.6% of IMG/M 16S rRNA gene sequences in various orders), as well as plant-associated (particularly rhizosphere) environments, although this could partly be attributed to sampling bias of these globally distributed and immensely important ecosystems (Figure 7a). On the other hand, a paucity of Binatota-affiliated sequences was observed in marine settings, with sequences absent or minimally present for Binatales, HRBin30, UBA9968, and UTPRO1 data sets (Figure 7a). The majority of sequences from marine origin were sediment-associated, being encountered in hydrothermal vents, deep marine sediments, and coastal sediments, with only the Bin18 sequences sampled from IMG/M showing representation in the vast, relatively well-sampled pelagic waters (Figure 7d).

10.1128/mBio.00985-21.6 FIG S5 (A) Maximum-likelihood phylogenetic tree based on the 16S rRNA gene representatives from six Binatota orders with representative hit sequences (number of sequences in parentheses following the order name) from the IMG and NCBI-nt databases identified by Blastn. Orders are color coded following the color scheme in Fig. 1, and the number of hits from each database is shown in parentheses. Bootstrap value (from 100 bootstraps) is shown for branches with >70% support. (B to G) Ecological distribution of Binatota-affiliated 16S rRNA sequences. Representative 16S rRNA gene sequences from six out of the seven Binatota orders (order UBA12105 genome assembly did not contain a 16S rRNA gene) were searched against Integrated Microbial Genomes & Microbiomes (IMG/M) 16S rRNA public assembled metagenomes database using Blastn and the criteria specified in Materials and Methods. Binatota orders are shown on the *x* axis, while percent abundances in different environments (classified based on the GOLD ecosystem classification scheme) are shown on the *y* axis (B). Further subclassifications for each environment are shown for (C) terrestrial, (D) freshwater, (E) marine, (F) host-associated, and (G) engineered environments. Details, including GenBank accession number of hit sequences, are shown in Extended Data 3. Download FIG S5, PDF file, 0.4 MB. Copyright © 2021 Murphy et al. 2021 Murphy et al. https://creativecommons.org/licenses/by/4.0/ This content is distributed under the terms of the Creative Commons Attribution 4.0 International license.

In addition to the 16S rRNA-based analysis, we queried the data sets from which a Binatota MAG was binned using the sequence of their ribosomal protein S3 and estimating the Binatota relative abundance as the number of reads mapped to contigs with a Binatota ribosomal protein S3 as a percentage of the number of reads mapped to all contigs encoding a ribosomal protein S3 gene. Results showed relative abundances ranging between 0.1 and 10.21% (average 3.84 ± 3.21%) (Table S1).

In addition to phylum-wide patterns, order-specific environmental preferences were also observed. For example, in order Bin18, one of the two available genomes originated from the Mediterranean sponge Aplysina aerophoba. Analysis of the 16S rRNA data set suggests a notable association between Bin18 and sponges, with relatively high host-associated sequences (Figure 7a), the majority of which (58.3% NCBI-nt, 25.0% IMG/M) were recovered from the Porifera microbiome (Fig. 7e; Fig. S5f). Bin18-affiliated 16S rRNA gene sequences were identified in a wide range of sponges from 10 genera and 5 global habitat ranges (the Mediterranean genera *Ircinia*, *Petrosia*, *Chondrosia*, and *Aplysina*, the Caribbean genera *Agelas*, *Xestospongia*, and *Aaptos*, the Indo-West Pacific genus *Theonella*, the Pacific Dysideidae family, and the Great Barrier Reef genus *Rhopaloeides*), suggesting its widespread distribution beyond a single sponge species. The absolute majority of order Binatales sequences (83.0% NCBI-nt, 91.6% IMG/M) were of a terrestrial origin (Figure 7a; Fig. S5c), in addition to multiple rhizosphere-associated samples (7.5% NCBI-nt and 2.8% IMG/M, respectively) (Figure 7a; Fig. S5f). Notably, a relatively large proportion of Binatales soil sequences originated from either wetlands (peats, bogs) or forest soils (Fig. 7b; Fig. S5c), strongly suggesting the preference of the order Binatales to acidic and organic/methane-rich terrestrial habitats. This corresponds with the fact that 42 out of 48 Binatales genomes were recovered from soil, 38 of which were from acidic wetland or forest soils (Fig. 1; Table S1). Genomes of UBA9968 were recovered from a wide range of terrestrial and nonmarine aquatic environments, and the observed 16S rRNA gene distribution verifies their ubiquity in all but marine habitats (Fig. 7a; Fig. S5b to g). Finally, while genomes from orders HRBin30, UBA1149, and UTPRO1 were recovered from limited environmental settings (thermal springs for HRBin30, gaseous hydrocarbon impacted habitats, e.g., marine hydrothermal vents and gas-saturated Lake Kivu, for UBA1149, and soil and hydrothermal environments for UTPRO1) (Fig. 1; Table S1), 16S rRNA gene analysis suggested their presence in a wide range of environments from each macro-scale environment classification (Fig. 7a; Fig. S5b to g).

## DISCUSSION

### Expanding the world of methylotrophy.

The current study expands the list of lineages potentially capable of methylotrophy. An extensive repertoire of genes and pathways mediating the oxidation of multiple C_1_ compounds to formaldehyde (Fig. 2 and 4), formaldehyde oxidation to CO_2_ (Fig. S2), and formaldehyde assimilation pathways (Fig. S2) was identified, indicating that such capacity is a defining metabolic trait in the Binatota. A certain degree of order-level substrate preference was observed, with potential utilization of methanol in all orders except HRBin30, methylamine in all orders except UBA9968, S-containing C_1_ compound in Bin18, Binatales, and UBA9968, halogenated methane in Bin18, and possible methane utilization (methanotrophy) in Bin18 and Binatales (Figure 2a).

Aerobic methylotrophy has been documented in members of the alpha, beta, and gamma *Proteobacteria* (39), *Bacteroidetes* (40), *Actinobacteria* (e.g., genera *Arthrobacter* and *Mycobacterium*), *Firmicutes* (e.g., Bacillus methanolicus) (41), *Verrucomicrobia* (42), and “*Candidatus* Methylomirabilis” (NC10) (43). Further, studies employing genome-resolved metagenomics identified some signatures of methylotrophy, e.g., methanol oxidation (7, 44), formaldehyde oxidation/assimilation (45), and methylamine oxidation (7), in the *Gemmatimonadetes*, “*Candidatus* Rokubacteria,” *Chloroflexi*, *Actinobacteria*, *Acidobacteria*, and “*Ca.* Lambdaproteobacteria.” The possible contribution of Binatota to methane oxidation (methanotrophy) is especially notable, given the global magnitude of methane emissions and the relatively narrower range of organisms (*Proteobacteria*, *Verrucomicrobia*, and “*Candidatus* Methylomirabilis” [NC10]) (46) capable of this special type of methylotrophy. As described above, indirect evidence exists for the involvement of Binatota harboring TUSC-type CuMMO sequences in methane oxidation, while it is currently uncertain whether Binatota harboring SAR324/*Actinobacteria*-type CuMMO sequences are involved in oxidation of methane, gaseous alkanes, or both. pMMO of methanotrophs is also capable of oxidizing ammonia to hydroxylamine, which necessitates methanotrophs to employ hydroxylamine detoxification mechanisms (47). All 11 Binatota genomes encoding CuMMO also carried at least one homologue of *nir*, *nor*, and/or *nos* genes that could potentially convert harmful N-oxide byproducts to dinitrogen.

As previously noted (19), methylotrophy requires the possession of three metabolic modules: C_1_ oxidation to formaldehyde, formaldehyde oxidation to CO_2_, and formaldehyde assimilation. Within the world of methylotrophs, a wide array of functionally redundant enzymes and pathways have been characterized that mediate various reactions and transformations in such modules. In addition, multiple combinations of different modules have been observed in methylotrophs, with significant variations existing even in phylogenetically related organisms. Our analysis demonstrates that such metabolic versatility indeed occurs within the methylotrophic modules of Binatota. While few phylum-wide characteristics emerged, e.g., utilization of serine pathway for formaldehyde assimilation, absence of H_4_MPT-linked formaldehyde oxidation, and potential utilization of PEP carboxykinase (*pckA*) rather than PEP carboxylase (*ppc*) for CO_2_ entry to the serine cycle, multiple order-specific differences were observed, e.g., XoxF-type methanol dehydrogenase encoded by Bin18 and Binatales genomes, MDH2-type methanol dehydrogenase encoded by UBA1149 genomes, absence of methanol dehydrogenase homologues in HRBin30 genomes, absence of methylamine oxidation in order UBA9968, and potential utilization of the ethylmalonyl-CoA pathway for glyoxylate regeneration by the majority of the orders versus the glyoxylate shunt by UBA9968.

### Alkane degradation in the Binatota.

A second defining feature of the phylum Binatota, in addition to methylotrophy, is the widespread capacity for aerobic alkane degradation, as evident by the extensive arsenal of genes mediating aerobic degradation of short- (*prmABC*, propane monooxygenase), medium- (*alkB*, cyp153), and long-chain alkanes (*ladA*) identified (Fig. 3), in addition to complete pathways for odd- and even-numbered fatty acids oxidation (Fig. S3). Hydrocarbons, including alkanes, have been an integral part of the earth biosphere for eons, and a fraction of microorganisms have evolved specific mechanisms (O_2_-dependent hydroxylases and monooxygenases, anaerobic addition of fumarate) for their activation and conversion to central metabolites (48). Aerobic alkane-degradation capacity has so far been encountered in the *Actinobacteria*, *Proteobacteria*, *Firmicutes*, and *Bacteroidetes*, as well as in a few *Cyanobacteria* (48). As such, this study adds to the expanding list of phyla capable of aerobic alkane degradation.

### Metabolic traits explaining niche preferences in the Binatota.

Analysis of 16S rRNA gene data sets indicated that the Binatota display phylum-wide (preference to terrestrial habitats and methane/hydrocarbon-impacted habitats and rarity in pelagic marine environments) as well as order-specific (Bin18 in sponges, HRBin30 and UBA1149 in geothermal settings, Binatales in peats, bogs, and forest soils) habitat preferences (Fig. 7; Fig. S5). Such distribution patterns could best be understood in light of the phylum’s predicted metabolic capabilities. Soils represent an important source of methane, generated through microoxic and anoxic niches within soil’s complex architecture (49). Methane emission from soil is especially prevalent in peatlands, bogs, and wetlands, where incomplete aeration and net carbon deposition occurs. Indeed, anaerobic (50), fluctuating (51), and even oxic (52) wetlands represent one of the largest sources of methane emissions to the atmosphere. As well, terrestrial ecosystems represent a major source of global methanol emissions (53), with the release of methanol mediated mostly by demethylation reactions associated with pectin and other plant polysaccharides degradation. C_1_-metabolizing microorganisms significantly mitigate methane and methanol release to the atmosphere from terrestrial ecosystems (54), and we posit that members of the Binatota identified in soils, rhizosphere, and wetlands contribute to such a process. The special preference of order Binatales to acidic peats, bogs, forests, and wetlands could reflect a moderate acidophilic specialization for this order and suggest their contribution to the process in these habitats.

Within the phylum Binatota, it appears that orders HRBin30 and UBA1149 are abundant in thermal vents, thermal springs, and thermal soils, suggesting a specialization to high-temperature habitats (Fig. 7). The presence of Binatota in such habitats could be attributed to high concentrations of alkanes typically encountered in such habitats. Hydrothermal vents display steep gradients of oxygen in their vicinity, emission of high levels of methane and other gaseous alkanes, and thermogenic generation of medium- and long-chain alkanes (55). Indeed, the presence and activity of aerobic hydrocarbon degraders in the vicinity of hydrothermal vents have been well established (27, 28, 56).

The recovery of Binatota genomes from certain lakes could be a reflection of the high gaseous load in such lakes. Multiple genomes and a large number of Binatota-affiliated 16S rRNA sequences were binned and identified from Lake Kivu, a meromictic lake characterized by unusually high concentrations of methane (57). Biotically, methane evolving from Lake Kivu is primarily oxidized by aerobic methanotrophs in surface waters (57–59), and members of the Binatota could contribute to this process. Binatota genomes were also recovered from sediments in Lake Washington, a location that has long served as a model for studying methylotrophy (60, 61). Steep counter gradients of methane and oxygen occurring in the lake’s sediments enable aerobic methanotrophy to play a major role in controlling methane flux through the water column (62–65).

Finally, the occurrence and apparent wide distribution of members of the Binatota in sponges, particularly order Bin18, are notable and could possibly be viewed in terms of the wider symbiotic relationship between sponges and their microbiome. Presence of hydrocarbon degraders (66, 67), including methanotrophs (68), in the sponge microbiome has previously been noted, especially in deep-water sponges, where low levels of planktonic biomass restrict the amount of food readily acquired via filter feeding and hence biomass acquisition via methane and alkane oxidation is especially valuable.

### Carotenoid pigmentation: occurrence and significance.

The third defining feature of the Binatota, in addition to aerobic methylotrophy and alkane degradation, is the predicted capacity for carotenoid production. In photosynthetic organisms, carotenoids increase the efficiency of photosynthesis by absorbing in the blue-green region and then transferring the absorbed energy to the light-harvesting pigments (69). Carotenoid production also occurs in a wide range of nonphotosynthetic bacteria belonging to the *Alphaproteobacteria*, *Betaproteobacteria*, and *Gammaproteobacteria* (including methano- and methylotrophs) and *Bacteroidetes*, *Deinococcus*, *Thermus*, Deltaproteobacteria, *Firmicutes*, *Actinobacteria*, *Planctomycetes*, and *Archaea*, e.g., *Halobacteriaceae* and *Sulfolobus*. Here, carotenoids could serve as antioxidants (70) and aid in radiation, UV, and desiccation resistance (71, 72). The link between carotenoid pigmentation and methylo/methanotrophy has long been observed (73), with the majority of known model *Alphaproteobacteria* and *Gammaproteobacteria* methano- and methylotrophs being carotenoid producers, although several Gram-positive methylotrophs (Mycobacterium, *Arthrobacter*, and *Bacillus*) are not pigmented. Indeed, root-associated facultative methylotrophs of the genus *Methylobacterium* have traditionally been referred to as “pink-pigmented facultative methylotrophs” and are seen as an integral part of root ecosystems (74). The exact reason for this correlation is currently unclear and could be related to the soil environment where they are prevalent, where periodic dryness and desiccation could occur, or to the continuous exposure of these aerobes in some habitats to light (e.g., in shallow sediments), necessitating protection from UV exposure.

### Chlorophyll biosynthesis genes in the Binatota.

Perhaps the most intriguing finding in this study is the identification of the majority of genes required for the biosynthesis of bacteriochlorophylls from protoporphyrin IX (6 out of 10 genes for bacteriochlorophyll *a* and 7 out of 11 genes for bacteriochlorophyll *c* and *d*). While such a pattern is tempting to propose phototrophic capacities in the Binatota, the consistent absence of critical genes (*bchM* methyltransferase, *bciA*/*bciB*/*bchXYZ* reductases, *bciC* hydrolase, and *bchF*/*V* hydratases), coupled with our inability to detect reaction center-encoding genes, prevents such a proclamation. Identification of a single or few gene shrapnel from the chlorophyll biosynthesis pathway in microbial genomes is not unique. Indeed, searching the functionally annotated bacterial tree of life AnnoTree (75) using single KEGG orthologies implicated in chlorophyll biosynthesis identifies multiple hits (in some cases thousands) in genomes from nonphotosynthetic organisms (Figure 6c). This is consistent with the identification of a *bchG* gene in a “*Candidatus* Bathyarchaeota” fosmid clone (76) and, more recently, a few bacteriochlorophyll synthesis genes in an Asgard genome (77). However, it should be noted that the high proportion of genes in the bacteriochlorophyll biosynthetic pathway identified in the Binatota genomes has never previously been encountered in nonphotosynthetic microbial genomes. Indeed, a search in AnnoTree for the combined occurrence of all seven bacteriochlorophyll synthesis genes identified in Binatota genomes yielded only photosynthetic organisms.

Accordingly, we put forward three scenarios to explain the proposed relationship between Binatota and phototrophy. The most plausible scenario, in our opinion, is that members of the Binatota are pigmented nonphotosynthetic organisms capable of carotenoid production but incapable of chlorophyll production and lack a photosynthetic reaction center. The second scenario posits that members of the Binatota are indeed phototrophs, possessing a complete pathway for chlorophyll biosynthesis and a novel type of reaction center that is bioinformatically unrecognizable. A minimal photosynthetic electron transport chain, similar to that of Chloroflexus aurantiacus (78), with the yet-unidentified reaction center, quinone, alternate complex III (or complex III), and some type of cytochrome c would possibly be functional. Under such scenario, members of the Binatota would be an extremely versatile photoheterotrophic facultative methylotrophic lineage. While such versatility, especially coupling methylotrophy to phototrophy, is rare (79), it has previously been observed in some *Rhodospirillaceae* species (80). A third scenario is that Binatota are capable of chlorophyll production but still incapable of conducting photosynthesis. Under this scenario, genes being missed in the pathway is due to shortcomings associated with *in silico* prediction and conservative gene annotation. For example, the missing *bchM* (EC: 2.1.1.11) could possibly be encoded by general methyltransferases (EC: 2.1.1.-), the missing *bciC* (EC: 3.1.1.100) could possibly be encoded by general hydrolases (EC: 3.1.1.-), and the missing *bchF* (EC: 4.2.1.165) or *bchV* (EC: 4.2.1.169) could possibly be encoded by general hydratases (EC: 4.2.1.-).

Encountering incomplete pathways in genomes of uncultured lineages is an exceedingly common occurrence in SAG and MAG analysis (81, 82). In many cases, this could plausibly indicate an incomplete contribution to a specific biogeochemical process, e.g., incomplete denitrification of nitrate to nitrite but not ammonia (82) or reduction of sulfite but not sulfate to sulfide (83), provided the thermodynamic feasibility of the proposed partial pathway and, preferably, prior precedence in pure cultures. In other cases, a pattern of absence of peripheral steps could demonstrate the capability for synthesis of a common precursor, e.g., synthesis of precorrin-2 from uroporphyrinogen but lack of the peripheral pathway for corrin ring biosynthesis leading to an auxotrophy for vitamin B_12_. Such auxotrophies are common in the microbial world and could be alleviated by nutrient uptake from the outside environment (84) or engagement in a symbiotic lifestyle (85). However, arguments for metabolic interdependencies, syntrophy, or auxotrophy could not be invoked to explain the consistent absence of specific genes in a dedicated pathway, such as bacteriochlorophyll biosynthesis, especially when analyzing a large number of genomes from multiple habitats. As such, we here raise awareness that using a certain occurrence threshold to judge a pathway’s putative functionality could lead to misinterpretations of organismal metabolic capacities due to the frequent occurrence of partial, nonfunctional pathways and “gene shrapnel” in microbial genomes.

In conclusion, our work provides a comprehensive assessment of the yet-uncultured phylum Binatota and highlights its aerobic methylotrophic and alkane-degradation capacities, as well as its carotenoid production and abundance of bacteriochlorophyll synthesis genes in its genomes. Future efforts should focus on confirming these *in silico* predicted capabilities and characteristics through targeted enrichment and isolation efforts as well as functional genomics approaches. We also propose a role for this lineage in mitigating methanol and perhaps even methane emissions from terrestrial and freshwater ecosystems, alkanes degradation in hydrocarbon-rich habitats, and nutritional symbiosis with marine sponges. We present specific scenarios that could explain the unique pattern of chlorophyll biosynthesis gene occurrence and stress the importance of detailed analysis of pathways completion patterns for appropriate functional assignments in genomes of uncultured taxa.

## MATERIALS AND METHODS

### Genomes.

All genomes classified as belonging to the Binatota in the Genome Taxonomy Database (GTDB) database (*n* = 22 MAGs, April 2020) were downloaded as assemblies from NCBI. In addition, 128 metagenome-assembled genomes with the classification “Bacteria;UBP10” were downloaded from the IMG/M database (April 2020). These genomes were recently assembled from public metagenomes as part of a wider effort to generate a genomic catalogue of Earth’s microbiome (13). Finally, 6 metagenome-assembled genomes were obtained as part of the Microbial Dark Matter MDM-II project. CheckM (86) was utilized for estimation of genome completeness, strain heterogeneity, and genome contamination. Only genomes with >70% completion and <10% contamination (*n* = 108) were retained for further analysis (Tables S1 and S2). MAGs were classified as high- or medium-quality drafts based on the criteria set forth by reference 15. The utilization of all publicly available genomes through prior individual efforts, as well as the global comprehensive Earth Microbiome collection, ensures the global scope of the survey conducted. Continuous addition of new data sets would certainly increase the number of available high-quality Binatota MAGs in the future.

### Phylogenetic analysis.

Taxonomic classifications followed the Genome Taxonomy Database (GTDB) release r89 (11, 87) and were carried out using the classify_workflow in GTDB-Tk (88) (v1.1.0). Phylogenomic analysis utilized the concatenated alignment of a set of 120 single-copy marker genes (11, 87) generated by the GTDB-Tk. Maximum-likelihood phylogenomic tree was constructed in RAxML (89) (with a cultured representative of the phylum Deferrisomatota as the outgroup). Small-subunit (SSU) rRNA gene-based phylogenetic analysis was also conducted using 16S rRNA gene sequences extracted from genomes using RNAmmer (90). Putative taxonomic ranks were deduced using average amino acid identity (AAI; calculated using AAI calculator [http://enve-omics.ce.gatech.edu/]), with the arbitrary cutoffs 56% and 68% for family and genus, respectively.

### Annotation.

Protein-coding genes in genomic bins were predicted using Prodigal (91). For initial prediction of function, pangenomes were constructed for each order in the phylum Binatota separately using PIRATE (92) with percent identity thresholds of 40, 45, 50, 55, 60, 65, 70, 75, 80, 90, a cd-hit step size of 1, and CD-HIT lowest percent identity of 90. The longest sequence for each PIRATE-identified allele was chosen as a representative and assembled into a pangenome. These pangenomes were utilized to gain preliminary insights on the metabolic capacities and structural features of different orders. BlastKOALA (93) was used to assign protein-coding genes in each of the pangenomes constructed to KEGG orthologies (KO), which were subsequently visualized using KEGG mapper (94). Analysis of specific capabilities and functions of interest was conducted on individual genomic bins by building and scanning hidden Markov model (HMM) profiles. All predicted protein-coding genes in individual genomes were searched against custom-built HMM profiles for genes encoding C_1_, alkanes, and fatty acids metabolism, C_1_ assimilation, [NiFe] hydrogenases, electron transport chain complexes, and carotenoid and chlorophyll biosynthesis. To build the HMM profiles, Uniprot reference sequences for all genes with an assigned KO number were downloaded and aligned using Clustal-omega (95), and the alignment was used to build an HMM profile using hmmbuild (HMMER 3.1b2). For genes not assigned a KO number (e.g., alternative complex III genes, different classes of cytochrome c family, cytochrome P450 medium-chain alkane hydroxylase cyp153, methanol dehydrogenase MNO/MDO family), a representative protein was compared against the KEGG Genes database using BLASTP and significant hits (those with E values <e−80) were downloaded and used to build HMM profiles as explained above. The custom-built HMM profiles were then used to scan the analyzed genomes for significant hits using hmmscan (HMMER 3.1b2) with the option -T 100 to limit the results to only those profiles with an alignment score of at least 100. Further confirmation was achieved through phylogenetic assessment and tree building procedures, in which potential candidates identified by hmmscan were aligned to the reference sequences used to build the custom HMM profiles using Clustal-omega (95), followed by maximum-likelihood phylogenetic tree construction using FastTree (96). Only candidates clustering with reference sequences were deemed true hits and were assigned to the corresponding KO.

### Search for photosynthetic reaction center.

Identification of genes involved in chlorophyll biosynthesis in Binatota genomes prompted us to search the genomes for photosynthetic reaction center genes. HMM profiles for reaction center type 1 (RC1; PsaAB) and reaction center type 2 (RC2; PufLM and PsbD_1_D_2_) were obtained from the pfam database (pfam00223 and pfam00124, respectively). Additionally, HMM profiles were built for PscABCD (*Chlorobia*-specific), PshA/B (*Heliobacteria*-specific) (97), and the newly identified Psa-like genes from *Chloroflexota* (98). The HMM profiles were used to search Binatota genomes for potential hits using hmmscan. To guard against overlooking a distantly related reaction center, we relaxed our homology criteria (by not including -T or -E options during the hmmscan). An additional search using a structurally informed reaction center alignment (97, 99) was also performed. The best potential hits were modeled using the SWISS-MODEL homology modeler (100) to check for veracity. Since the core subunits of type 1 RC proteins are predicted to have 11 transmembrane α-helices, while type 2 RC are known to contain 5 transmembrane helices (101, 102), we also searched for all predicted proteins harboring either 5 or 11 transmembrane domains using TMHMM (103). All identified 5- or 11-helix-containing protein-coding sequences were searched against GenBank protein nr database to identify and exclude all sequences with a predicted function. All remaining 5- or 11-helix-containing proteins with no predicted function were then submitted to SWISS-MODEL homology modeler using the automated mode to predict homology models.

### Classification of [NiFe] hydrogenase sequences.

All sequences identified as belonging to the respiratory O_2_-tolerant H_2_-uptake [NiFe] hydrogenase large subunit (HyaA) were classified using the HydDB web tool (104).

### Particulate methane monooxygenase 3D model prediction and visualization.

SWISS-MODEL (100) was used to construct pairwise sequence alignments of predicted Binatota particulate methane monooxygenase with templates from Methylococcus capsulatus strain Bath (PDB: 3RGB) and to predict tertiary structure models. Predicted models were superimposed on the template enzyme in PyMol (version 2.0, Schrödinger, LLC). Modeling of the active site was conducted similarly. The dicopper-binding site proposed for Methylococcus capsulatus strain Bath pMMO (105) (PDB: 3RGB) was used. Alignment of Binatota PmoB sequences with reference Methylococcus capsulatus strain Bath PmoB was performed with Clustal-omega (95) and visualized using the ENDscript server (106).

### Ecological distribution of Binatota.

We queried 16S rRNA sequence databases using representative 16S rRNA gene sequences from six out of the seven Binatota orders (order UBA12105 genome assembly did not contain a 16S rRNA gene). Two databases were searched: (i) GenBank nucleotide (nt) database (accessed in July 2020) using a minimum identity threshold of 90%, ≥80% subject length alignment for near full-length query sequences or ≥80% query length for non-full-length query sequences, and a minimum alignment length of 100 bp and (ii) The IMG/M 16S rRNA public assembled metagenomes using a cutoff E value of 1e−10, percentage similarity of ≥90%, and either ≥80% subject length for full-length query sequences or ≥80% query length for non-full-length query sequences. Hits satisfying the above criteria were further trimmed after alignment to the reference sequences from each order using Clustal-omega and inserted into maximum-likelihood phylogenetic trees in FastTree (v 2.1.10, default settings). The ecological distribution for each of the Binatota orders was then deduced from the environmental sources of its hits. All environmental sources were classified according to the GOLD ecosystem classification scheme (107). We also queried the data sets from which the Binatota MAGs were binned using the sequence of their ribosomal protein S3. We estimated their relative abundance as the number of reads mapped to contigs with a Binatota ribosomal protein S3 as a percentage to the number of reads mapped to all contigs carrying a ribosomal protein S3 gene. More details on the specifics of the search are in Table S1 footnotes.

### Data availability.

Genomic bins, predicted proteins, and extended data for Fig. 2, 3, 5, and 6, Fig. S2 to S4, Fig. 7a to f, and Fig. S5b to g are available at https://github.com/ChelseaMurphy/Binatota. Maximum-likelihood trees (Fig. 1 and Fig. S5a) can be accessed at https://itol.embl.de/shared/1WgxEjrQfEYWk. Maximum-likelihood trees for chlorophyll biosynthesis genes are available at https://itol.embl.de/shared/34y3BUHcQd7Lh.
