# Supplementary material for: Genomic Analysis of the Yet-Uncultured Binatota Reveals Broad Methylotrophic, Alkane-Degradation, and Pigment Production Capacities
Source: mBio. 2021 May 18;12(3):e00985-21. doi: 10.1128/mBio.00985-21 (PMC8262859; doi:10.1128/mBio.00985-21)

```
3RGB                                                         α1        β1
                   1        10        20        30        40        50
3RGB               MKTIKDRIAKWSAIGLLSAVAATAFYAP.SASAHGEKSQAAFMRMRTIHWYDLSWSK
3300005529_54_1    ..MARQRLI......LILAFILTAAIAPASAFAHGEAGDEPFLKDLTTAFYDVKITP
3300005529_28_1    ..MDKRIVT......LVLALTLAVTVSVSKVWAHGEAGDQPFLKNMTTAFYDVSISP
3300017426_14_1    ..MRKWFAI......SIPAILLLCLSVKS.ACAHGEAADEPFLKDLTVAFYNVHVSP
3300027968_51_1    MEHRKRAMSAGGMIAALAAVIGAILGAAGSAAAHGERAQEPSLRMLTVQFYDTKWST
3300027965_49_1    MEHRKRAMSAGGMIAALAAVIGAILGAAGSAAAHGERAQEPSLRMLTVQFYDTKWST
3300027706_20_1    .MTKKRRLT......WIAVLAIAAFIAPSKAFAHGEAGDEPFLKDLTTAFYDVSISP
3300027773_28_1    ..MRRWLLS......GILTLLVTCFSVAQ.AFAHGEAADEPFLKDLTVAFYNVHVSP
MDM2_1362_1        ..MRIRFPV......LSAALLILIASYAPRAFAHGEAADEPFLKDLTVAFYDVHISP
MDM2_1401_1        ..MRKWFAI......SIPAILLLCLSVKS.ACAHGEAADEPFLKDLTVAFYNVHVSP
GCA_002238415.1    ..MSPLGKACLAVSLTCLCVFGVSFMQAPVVQAHGEHNLEPFIRMRGISFFNVEFSH
GCA_003135855.1    ..MRKRSLVGV..VAAIAALAIATIFGVPKAFAHGEAGDEPFLKDLTTAFYDVHISP
consensus>50       ..................a...........a.AHGE..depfl..lt..f%#v..s.

3RGB                β2    β3               β4         β5      β6
                    60        70        80        90        100       110
3RGB               EKVKINETVEIKGKFHVFEGWPETVDEPDVAFLNVGMPGPVFIRKESYIGGQLVPRS
3300005529_54_1    NVIQVGEPVTITGTVRVLDTWPYTLDPPETAYITPVVPGPVFLLKDRIVNGQAAPGS
3300005529_28_1    AEVEVGQPVTVTGKVKVLETWPQTLKDPDIAAIVPVVPGPVFALKDRTINGQPAIGS
3300017426_14_1    TNIRVGDPVTVTGSLKILETWPYTLDPPQTAYITPVVPGPVFALVDRVINGQPAFGS
3300027968_51_1    DKVPVNGEITVSGRFHVMKNWPLEIYEPGLAYLNVAVPGPVFVRVASSIGEVNGFAS
3300027965_49_1    DKVPVNGEITVSGRFHVMKNWPLEIYEPGLAYLNVAVPGPVFVRVASSIGEVNGFAS
3300027706_20_1    KAIHVGEPVTITGTVRILDTWPYTLDPPQTAYITPVVPGPVFALEDRVINGQPAPGS
3300027773_28_1    TSITVGQPVTITGSLKILETWPYTLDPPVTAYITPVVPGPVFALVDREVNGQPAFGS
MDM2_1362_1        TTIKVGDPVTITGTVKILETWPYTLDPPQTAYITPVVPGPVFALVDRVVNGTTTPGS
MDM2_1401_1        TNIRVGDPVTVTGSLKILETWPYTLDPPQTAYITPVVPGPVFALVDRVINGQPAFGS
GCA_002238415.1    ERLAVNETMTVTGKFRAMNSWPRMLTTPELAWVGVLVPGPKLAVRERWVNGRFIQNA
GCA_003135855.1    TEIAVGQPVTITGTVRILETWPYTLYPPEKAYITPVVPGPVFVLRERTVNGQQAPGS
consensus>50       ..i.!.e.vt!tG...ile.WP.tl..Pq.Ayi...vPGPvf...dr.!ng.....s

3RGB                  TT    β7          β8           β9           η1
                      120       130       140       150       160
3RGB               VRLEIGKTYDFRVVLKARRPGDWHVHTMMNVQGGGPIIGPGKWITVEGS..MSEFRN
3300005529_54_1    FFVQRGGVYQFKMVIVGREPGRWHVHPGIAVQGTGTLLGPGEWVDVRPS..AANFTF
3300005529_28_1    FFAEKGGIYEFKMTLLGKEPGKWHVHPGIAISGTGTLVGPGDWVTVKP...GGKFEF
3300017426_14_1    ITVERGGTYSFKMVMRGRNPGHWHVHPGIAIQGTGTLIGPGEWVTVEPS..ATPFKL
3300027968_51_1    VKLELGQEYEYKLVLKGRLPGRYHVHPMLDVQDVGPIVGPGKWVTVEAAPAQARFVD
3300027965_49_1    VKLELGQEYEYKLVLKGRLPGRYHVHPMLDVQDVGPIVGPGKWVTVEAAPAQARFVD
3300027706_20_1    FFVERGGVYQFKMVMVGREPGRWHVHPGIAVQGTGTLVGPGEWVDVQPA..VAKFTF
3300027773_28_1    ITVERGGTYDFKMVMRGRNPGRWHVHPGIAIQGTGTLIGPGEWVTVSPS..DTPFKL
MDM2_1362_1        ILVDKGRTYHFKMVMRARDPGNWHVHPGIAIQGTGTLIGPGEWVTVEPA..AEPFKM
MDM2_1401_1        ITVERGGTYSFKMVMRGRNPGHWHVHPGIAIQGTGTLIGPGEWVTVEPS..ATPFKL
GCA_002238415.1    FNIELGALYDFKMILQAREPGRYHVHPLINLSGTGGLVGPAKWITVVPG.DTPPSYT
GCA_003135855.1    FFVEKGGVYNFKMEILGRNPGKWHVHPGIAVQGTGTLIGPGEWVTVKPS..GAPFVF
consensus>50       ...#.G..Yd%kmvl.grePG.wHVHp.i.vqgtG.liGPg.W!tV.p......f..

3RGB               β10  TT β11          α2                      α3
                 170       180       190       200       210       220
3RGB               PVTTLTGQTVDLENYNEGNTYFWHAFWFAIGVAWIGYWSRRPIFIPRLLMVDAGRAD
3300005529_54_1    PVTLLSGTTINLEDYEGSFAWWWSFVGFLIGVGWMLYWTLSKRTVTNLAVTTQIGIN
3300005529_28_1    PVQLLNGQTIELNTYGGQFVWWWSFAGFLLGMAWMVYWTFTKRTVTNLAVTVQLSPN
3300017426_14_1    NVMLLNGQTVDLEHFGGSFVWWWSLAGFIIGLVWMIYWTVPKRTVTRLAVTSQLGAN
3300027968_51_1    PVTTLTGHTVDLASYGLGWVIKYHLVWLALGLAWLVYWIRKPMLMPRYEAVQSGEAD
3300027965_49_1    PVTTLTGHTVDLASYGLGWVIKYHLVWLALGLAWLVYWIRKPMLMPRYEAVQSGEAD
3300027706_20_1    PVTLLNGKVIDLEGYQSSFVWWWSFAGFLIGVAWMLYWTLSKRTVTNLAVTTQIGIN
3300027773_28_1    NVMLLNGTTVDLEHFGGGFVWWWSFAGFIVGLVWMIYWTVPKRTVTRLAVTSQLGAN
MDM2_1362_1        DVTLLTGQTVNLEHYGGSFVWWWSFAGFIIGVIWLLYWTVPKRTVTRLAVTTQLPVN
MDM2_1401_1        NVMLLNGQTVDLEHFGGSFVWWWSLAGFIIGLVWMIYWTVPKRTVTRLAVTSQLGAN
GCA_002238415.1    IQIPATGETIDLEHYGFGRVVGWHIAFGILGLAWLVYWARRPMLVRYALLVNGVDEE
GCA_003135855.1    PVKLLSGQTINLNKYEGGFVWWWSFAGFLIGVAWMLYWTLNKRTVTNLAVTIQLGVN
consensus>50       .v..l.G.t!#Le.%....v..w....f..G..Wm.YW......v..l........#

3RGB                              α4
                         230       240       250       260       270
3RGB               E......LVSATDRKVAMGFLAATILIVVMAMSSANSKYPITIPLQAGTMRGMKPLE
3300005529_54_1    EDAPDIGLITPRDQMWMNVFAGVTIVLLIAGWFYVAGRYPVRLPQQTDWFA.PRPLV
3300005529_28_1    DDAPDIGLITPRDHMWMNIITGLTVLLLIIGWTYAATSYPVRWPQQTDWIR.PAHIS
3300017426_14_1    DDAPDIGLITPEDHKWMNVLAGITIAMLVVGWVWMAAKYPVRLPQQTDRFA.PEVET
3300027968_51_1    Q......LITRGDRLAALVVLGASLMVAFGGLAIADSMFPLTIPLQSDWTR.AKPLP
3300027965_49_1    Q......LITRGDRLAALVVLGASLMVAFGGLAIADSMFPLTIPLQSDWTR.AKPLP
3300027706_20_1    EDAPDIGLITPRDQMWMNVLAGLTIALLILGWFYAATRYPVRLPQQTDWFA.PRPLE
3300027773_28_1    DDAPDIGLITREDHKWMNVLAAITILMLVGGWAWMAAKYPVRLPQQTDRFA.PTLDT
MDM2_1362_1        DDAPDIGLITPRDHMWMDLLAGATVAMLVIGWIYMAVKFPVRLPQQTVRFA.PPVID
MDM2_1401_1        DDAPDIGLITPEDHKWMNVLAGITIAMLVVGWVWMAAKYPVRLPQQTDRFA.PEVET
GCA_002238415.1    Q.......ITPTDRRTSLIFGVVLVVLVIGGLLVTNSGRPPIIPHQAARMWDLSGLP
GCA_003135855.1    DDAPDIGLITPRDHVWMNVLAGLTIAMLLIGWSYSAMKYPVRLPQQTDWFA.PQFVS
consensus>50       #......l!t..D.....v..g.ti.m.v.g.......yPv..P.Q.d.........
```

```
3RGB               β12 ....  β13  β14  β15
                   280        290       300       310       320

3RGB               LPAPTVSVKVEDA....TYRVPGRAMRMKLTITNHGNSPIRLGEFYTASVRFLDSDV
3300005529_54_1    ADEKLAEVEPMGA....SWNDGTDTLIMKVRAKNIGNAPITLKEFIMAMTTFVNGNA
3300005529_28_1    SGPVMAEVHTNGA....TYDDATDTLVMKVQVKNVADSAISVNRMIVGPATFVKGGE
3300017426_14_1    QKYHLAQVTPAGSPNNATYDEKTDTLTINLDVKNLAGSPITPKQYSMAMASFVNGSE
3300027968_51_1    ESSAEISVEPLAA....TYLVPGRSMQFKLLVTNNSHEAVKLGEFTTANLRFINSAV
3300027965_49_1    ESSAEISVEPLAA....TYLVPGRSMQFKLLVTNNSHEAVKLGEFTTANLRFINSAV
3300027706_20_1    PDLKLAEVRPEGA....TWNDGTDTLVMKVQAKNVSDSPITLKEYVMGMTTFVNGGP
3300027773_28_1    SSYHLAEVRPVGSPNNATFDEKTNTLTISLDVKNTGTQPIMLKQYSMAMATFVNGSQ
MDM2_1362_1        STEHAAQVMNADS....TYDEKTDTLLINVAVKNTGTAPLSLKQYIMAMATFVNGGK
MDM2_1401_1        QKYHLAQVTPAGSPNNATYDEKTDTLTINLDVKNLAGSPITPKQYSMAMASFVNGSE
GCA_002238415.1    IPSAVQTEVKELR.....YNTTERSLSIQVEATNASDQAVVLRKFTTSYLSFINPAT
GCA_003135855.1    SGEKMAEVSPQGA....TYDDGTDTLVMKVNVKNIGPSPITVKQYIMAMATFVNGKP
consensus>50       .......v.........tyd.....$.....v.N.....i...ey..a...Fvn...

3RGB               β16 TT β17   TT  β18  α5  η2
                   330       340       350       360       370       380

3RGB               YKDTTGYPEDLLAEDGLSVSDN.SPLAPGETRTVDVTASDAAWEVYRLSDIIYDPDS
3300005529_54_1    EAQAGAGPRDYVGR...LEVEPNVAIAPGETRELTLKITSPILSEERLIPTR.DPQQ
3300005529_28_1    QEQAKAGPREFVGS...LQVQPGESIAPGETKELTLTVESNIFSTVRLIPLR.DPQQ
3300017426_14_1    QEQANAGPRDFVGQ...LQVEPSGPIGTGETKRITLKMASPIFDEERLIPLH.DPQQ
3300027968_51_1    EAADSAYPAELIAPGGLVIKAGGDEIAPGQTKEVYFEATSPIWETDRLASLVRDPTN
3300027965_49_1    EAADSAYPAELIAPGGLVIKAGGDEIAPGQTKEVYFEATSPIWETDRLASLVRDPTN
3300027706_20_1    DAQAQAGPHEYVGQ...LEVEPNGPIAPGETKELTLRIANPILSEERLIPTR.DPQQ
3300027773_28_1    QELAEAGPHDFVGS...LQVEPPDPIPAGQTRRVVLKMASPIFDEERLIPLH.DPQQ
MDM2_1362_1        DDLDKAGPAHYVGS...MQVQPDAPIAPGQTGKMQLKISSPLFDEERLIPLH.DPQE
MDM2_1401_1        QEQANAGPRDFVGQ...LQVEPSGPIGTGETKRITLKMASPIFDEERLIPLH.DPQQ
GCA_002238415.1    QDLYSDLQNEIPTM....QVEPDGLFQPGETKMLTLTMQDAIWETDRFIEFD.QPQI
GCA_003135855.1    DDLAKAGPHDYVGQ...LEVDPDSPIAPGDAKDLTLRISNPVLSDERLIPLH.DPQQ
consensus>50       q....a.p.d.v......qvep...i.pG#t....l....pi...eRli....#Pqq

3RGB               β19 TT   β20  β21
                   390       400       410

3RGB               RFAGLLFFFDAT.............GNRQVVQIDAPLIPSFM...............
3300005529_54_1    FIAGLLRFQSAA.............GGEQFAVARVNVVPTQFKAQYIPGL.......
3300005529_28_1    FVGAVVQFQNAQ.............GGQQLVTVRSNVVPTQFRGQFLP.........
3300017426_14_1    LIAGLLAFRDGA.............GREEMVTVKSVLVPTEFKPQYLP.........
3300027968_51_1    RVGGLLMFYDSA.............GKRSVVEFSHEIIPTFTGPLANAY........
3300027965_49_1    RVGGLLMFYDSA.............GKRSVVEFSHEIIPTFTGPLANAY........
3300027706_20_1    FIAGLLRFEKAG.............GGEQFAVVRINVIPTQFKAQYIPGL.......
3300027773_28_1    LIAGLLAVRDNV.............GREERVTVKSVLVPTEFQAQYLP.........
MDM2_1362_1        RIAGLLRFTDAQ.............GHEDLVTLQTDLVPTEFRPQYLP.........
MDM2_1401_1        LIAGLLAFRDGA.............GREEMVTVKSVLVPTEFKPQYLP.........
GCA_002238415.1    TAAGVLIFTDAQPLAREGLQPIMMGNSREGLGVPWEHISSLNEVYSNIQIDFGSMGQ
GCA_003135855.1    FIAGLLRFENGL.............GKQEMVVIRSGVIPTQFKSQYLP.........
consensus>50       .iagll.f.d...............g..e.v......!pt.................
```

B

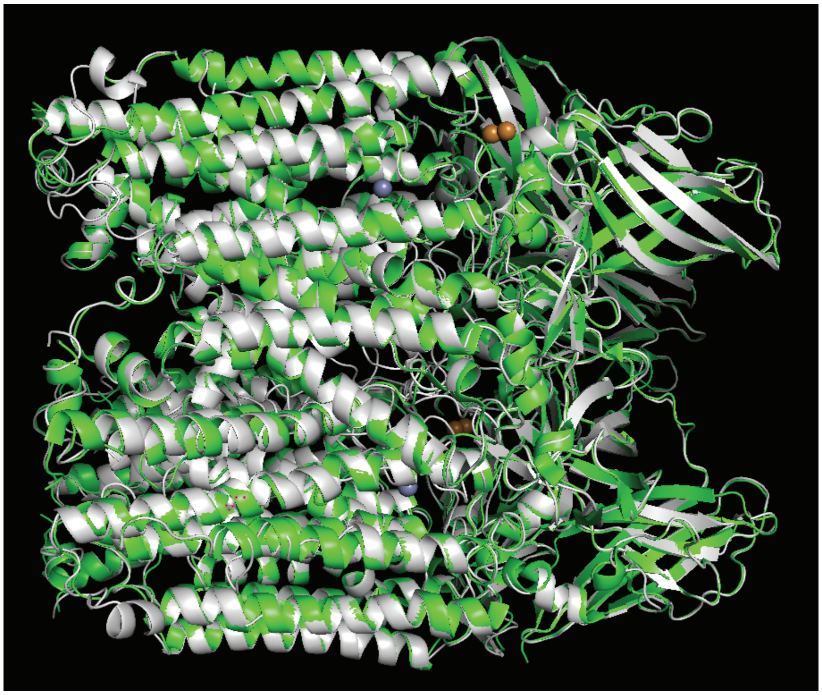

C

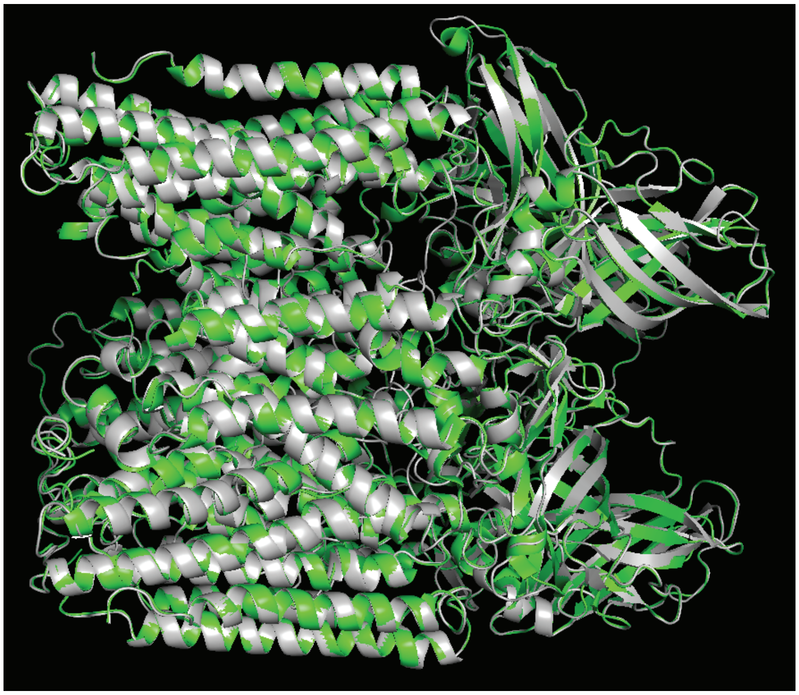

Supplement: FIG S1 [file mbio.00985-21-sf001.pdf]
