# Supplementary material for: Genomic Analysis of the Yet-Uncultured Binatota Reveals Broad Methylotrophic, Alkane-Degradation, and Pigment Production Capacities
Source: mBio. 2021 May 18;12(3):e00985-21. doi: 10.1128/mBio.00985-21 (PMC8262859; doi:10.1128/mBio.00985-21)
Supplement: TEXT S1 [file mbio.00985-21-s0001.pdf]

Supplementary data for:

Methylotrophy, alkane-degradation, and pigment production as defining features of the globally distributed yet-uncultured phylum Binatota

Chelsea L. Murphy[1], Andriy Sheremet[2], Peter F. Dunfield[2], John R. Spear[3], Ramunas Stepanauskas[4], Tanja Woyke[5], Mostafa S. Elshahed[1], and Noha H. Youssef[1]*

Supplementary text.

Formaldehyde oxidation to CO_2 by members of the Binatota: Three different routes for formaldehyde oxidation to formate were identified (Figure S2). First, the Actinobacteria specific thiol-dependent formaldehyde dehydrogenase (*fadh/mscR*) (EC: 1.1.1.306) was, surprisingly, detected in the majority (96 out of 108) of genomes (Figure S2a, Extended data 1). The enzyme requires a specific thiol (mycothiol (1)), the biosynthesis of which (encoded by *mshABC* gene cluster) was also encoded in Binatota genomes (Figure S2a). Second, the tetrahydrofolate (H_4F)-linked pathway comprising the genes *folD* (encoding bifunctional methylene-H_4F dehydrogenase and methenyl-H_4F cyclohydrolase) and either *ftfL* (the reversible formyl-H_4F ligase) or *purU* (the irreversible formyl-H_4F hydrolase) was also widespread (98/108 genomes). Finally, 40 genomes (Bin18, Binatales, HRBin30, and UTPRO1) also encoded the single gene/enzyme NAD-linked glutathione-independent formaldehyde dehydrogenase *fdhA*. Surprisingly, no evidence of the most common formaldehyde oxidation pathway (tetrahydromethanopterin (H_4MPT)-linked) was detected in any of the Binatota genomes. The NAD- and glutathione-dependent formaldehyde oxidation pathway was found incomplete: while homologs of formaldehyde dehydrogenase (*frmA*) were detected in almost all Binatota genomes, S-formylglutathione hydrolase (*frmB*) were absent. Following formaldehyde oxidation to formate, formate is subsequently oxidized to CO_2 by one of many formate dehydrogenases. The majority of Binatota genomes (103/108) encoded at least one copy of the NAD-dependent formate dehydrogenase (EC: 1.17.1.9) (Figure S2a, Extended data 1).

Formaldehyde assimilation by members of the Binatota. Two pathways for formaldehyde assimilation by methylotrophs have been described: the serine cycle, which assimilates 2 formaldehyde molecules and 1 CO_2 molecule, and the ribulose monophosphate cycle (RuMP),

which assimilates 3 formaldehyde and no CO_2 molecules. In addition, some methylotrophs assimilate carbon at the level of CO_2 via the Calvin Benson Bassham (CBB) cycle (2). Homologs encoding the RuMP cycle-specific enzymes were missing from all Binatota genomes, and only three genomes belonging to the Binatales order encoded the CBB cycle enzymes phosphoribulokinase and rubisCO. On the other hand, genes encoding enzymes of the serine cycle (Figure S2b) were identified in all genomes (Figure S2c, Extended data 1), with the key enzymes that synthesize and cleave malyl-CoA (*mtkA/B* [EC 6.2.1.9] malate-CoA ligase, and *mcl* [EC 4.1.3.24] malyl-CoA lyase, respectively) encountered in 98, and 86 Binatota genomes, respectively (Figure S2c, Extended data 1). The entry point of CO_2 to the serine cycle is the phosphoenolpyruvate (PEP) carboxylase (*ppc*) step catalyzing the carboxylation of PEP to oxaloacetate (Figure S2b). Homologues of *ppc* were missing from most Binatota genomes. Instead, all genomes encoded PEP carboxykinase (*pckA*) that replaces *ppc* function as shown in methylotrophic mycobacteria (3) (Figure S2b-c, Extended data 1).

During the serine cycle, regeneration of glyoxylate from acetyl-CoA is needed to restore glycine and close the cycle. Glyoxylate regeneration can be realized either through the classic glyoxylate shunt (4), or the ethylmalonyl-CoA pathway (EMCP) (5) (Figure S2b). All Binatota genomes exhibited the capacity for glyoxylate regeneration, but the pathway employed appears to be order-specific. Genes encoding all EMCP pathway enzymes were identified in genomes belonging to the orders Bin18, Binatales, HRBin30, UBA1149, and UBA12105 (Figure S2c, Extended data 1), including the two EMCP-specific enzymes ethylmalonyl-CoA mutase (*ecm*) and crotonyl-CoA reductase/carboxylase (*ccr*). On the other hand, order UBA9968 genomes lacked EMCP-specific enzymes but encoded the classic glyoxylate shunt enzymes isocitrate lyase (*aceA*) and malate synthase (*aceB*) (Figure S2c, Extended data 1).

Alkane degradation in the Binatota. Besides methylotrophy and methanotrophy, Binatota genomes exhibited extensive short-, medium-, and long-chain alkanes degradation capabilities. In addition to the putative capacity of Actinobacteria/SAR324-affiliated Cu-MMO to oxidize C_1-C_5 alkanes, and C_1-C_4 alkenes as described above, some Binatota genomes encoded propane-2-monoxygenase (*prmABC*), an enzyme mediating propane hydroxylation in the 2-position yielding isopropanol. Several genomes, also encoded medium chain-specific alkane hydroxylases, e.g. homologues of the nonheme iron *alkB* (6) and Cyp153-class alkane hydroxylases (7). The genomes also encoded multiple long-chain specific alkane monooxygenase, e.g. *ladA* homologues (EC:1.14.14.28) (8) (Figure 3, Extended data 1). Finally, Binatota genomes encoded the capacity to metabolize medium-chain haloalkane substrates. All genomes encoded *dhaA* (haloalkane dehalogenases [EC:3.8.1.5]) known to have a broad substrate specificity for medium chain length (C3 to C10) mono-, and dihaloalkanes, resulting in the production of their corresponding primary alcohol, and haloalcohols, respectively (9) (Figure 3, Extended data 1).

Alcohol and aldehyde dehydrogenases sequentially oxidize the resulting alcohols to their corresponding fatty acids or fatty acyl-CoA. Binatota genomes encode a plethora of alcohol and aldehyde dehydrogenases. These include the wide substrate range alcohol (EC:1.1.1.1), and aldehyde (EC:1.2.1.3) dehydrogenases encoded by the majority of Binatota genomes, as well as bifunctional alcohol/aldehyde dehydrogenase (EC:1.2.1.10 /1.1.1.1) encoded by a few Binatota genomes (7 genomes), and some highly specific enzymes, e.g. the short-chain isopropanol dehydrogenase (EC:1.1.1.80) for converting isopropanol and other secondary alcohols to the corresponding ketone (20 genomes), and acetone monooxygenase (*acmA*, EC:1.14.13.226) and

methyl acetate hydrolase (*acmB*, EC:3.1.1.114) that will sequentially oxidize acetone to methanol and acetate (6 genomes) (Figure 3, Extended data 1).

A Complete fatty acid degradation machinery that enables all orders of the Binatota to degrade short-, medium-, and long-chain fatty acids to acetyl CoA and propionyl-CoA were identified (Figure S3a, Extended data 1). Acetyl-CoA produced from the beta-oxidation pathway could be assimilated via the ethylmalonyl CoA pathway (EMCP) or the glyoxylate shunt as discussed above. Further, two pathways for propionyl-CoA assimilation, generated from the degradation of odd chain fatty acids, were identified (Figure S3b). Orders Bin18, Binatales, UBA1149, UBA12105, and UTPRO1 all encode enzymes for the methylmalonyl CoA (MMCoA) pathway that carboxylates propionyl CoA to succinyl-CoA (TCA cycle intermediate) via a methylmalonyl-CoA intermediate. On the other hand, the majority of order UBA9968 genomes encode enzymes of the 2-methylcitrate cycle for propionyl-CoA degradation (*prpBCD*) where propionate is degraded to pyruvate and succinate via a 2-methylcitrate intermediate (Figures S3b-c, Extended data 1).

Predicted electron transport chain. All Binatota genomes encode an aerobic respiratory chain comprising complexes I, II, and IV, as well as an F-type H^+-translocating ATP synthase (Figures 4, S4a, Extended data 1). Interestingly, genes encoding complex III (cytochrome bc1 complex) were sparse in Binatota genomes with some orders lacking genes encoding all subunits (e.g. HRBin30) and others only encoding the Fe-S (ISP) and the cytochrome b (*cytB*) but not the cytochrome c1 (*cyt1*) subunit (e.g. Binatales, UBA1149). Instead, genes encoding an Alternate Complex III (ACIII, encoded by *actABCDEFG*) were identified in 76 genomes, with 12 genomes encoding both complete complexes (in orders Bin 18, UBA9968, and UTPRO1). Complex III and ACIII transfer electrons from reduced quinones (all genomes encode the

capability of menaquinone biosynthesis) to cytochrome c which, in turn, reduces cytochrome c oxidase (complex IV). Homologues of the electron transfer proteins belonging to cytochrome c families were rare in Binatota genomes, especially those encoding ACIII (Figure S4a, Extended data 1). However, the recent structure of ACIII from *Flavobacterium johnsoniae* (10) in a supercomplex with cytochrome c oxidase aa3 suggests that electrons could potentially flow from ACIII to complex IV without the need for cytochrome c, which might explain the paucity of cytochrome c homologues in ACIII-harboring genomes.

Based on the predicted ETC structure, the flow of electrons under different growth conditions in the Binatota could be envisaged (Figure 4). When growing on methane, Cu-MMO would be coupled to the electron transport chain at complex III level via the quinone pool, where reduced quinones would act as physiological reductant of the enzyme (11) (Figure 4). Cu-MMO was also previously reported to receive electrons donated by NADH (12). During methanol oxidation by periplasmic enzymes (e.g. *xoxF*-type methanol dehydrogenases), and methylamine oxidation by the periplasmic methylamine dehydrogenase (*mauAB*) electrons would be shuttled via their respective C-type cytochrome (*xoxG*, and *mauC*, respectively) to complex IV. In the cytosol, methanol oxidation via the *mno/mdo*-type or the *mdh2*-type methanol dehydrogenases, as well as formaldehyde and formate oxidation via the action of cytoplasmic formaldehyde and formate dehydrogenases would contribute NADH to the aerobic respiratory chain through complex I. Similarly, when growing heterotrophically on alkanes and/or fatty acids, reducing equivalents in the form of NAD(P)H, and $FADH_2$ serve as electron donors for aerobic respiration through complex I, and II, respectively (Figure 4).

Binatota genomes also encode respiratory O_2-tolerant H_2-uptake [NiFe] hydrogenases, belonging to groups 1c (6 sequences), 1f (22 sequences), 1i (1 sequence), and 1h (4 sequences)

(Figure S4b). In *E. coli*, these membrane-bound periplasmically oriented hydrogenases transfer electrons (through their cytochrome b subunit) from molecular hydrogen to the quinone pool. Cytochrome *bd* oxidase (complex IV) then completes this short respiratory electron transport chain between H_2 and O_2 (13). In *E. coli*, the enzyme functions under anaerobic conditions (14), and may function as an O_2-protecting mechanism (15). Further, simultaneous oxidation of hydrogen (via type I respiratory O_2-tolerant hydrogenases) and methane (via Cu-MMO) has been shown to occur in methanotrophic Verrucomicrobia to maximize proton-motive force generation and subsequent ATP production (16). As well, some of the reduced quinones generated through H_2 oxidation are thought to provide reducing power for catalysis by Cu-MMO (16) (Figure 4).

Supplementary Tables:

Table S1. Binatota genomes used in this study#, their GTDB classification and the corresponding classification in Silva and RDP databases, the source from which they were obtained, as well as the calculated Binatota abundances in metagenomes with available contig coverage data.

Table S2. Sequencing statistics for the genomic bins used in this study

Table S3. General genomic features of the studied genomes.

Supplementary Figures:

Figure S1. (A) Alignment of the PmoB subunit of the 11 Copper membrane monooxygenases predicted in Binatota genomes to PmoB from Methylococcus capsulatus (pdb ID: 3RGB). The alignment is showing conserved residues (red highlight). Of particular importance, aer the three conserved histidine residues His_{33}, His_{137}, and His_{139}(shown with a blue rectangle), thought to coordinate the Cu cofactor. Numbering follows the Methylococcus capsulatus str. Bath PmoB subunit (pdb: 3RGB). Alignment was created using the ENDscript webserver (http://espript.ibcp.fr/ESPript/ESPript/). (B-C) Predicted Cu- methane monooxygenase (PmoABC) 3D structure (grey) from a Cluster 2 TUSC-affiliated Binatota genome (Genome 3300027968_51, (B)), and an Actinobacteria/SAR324-affiliated Binatota genome (Genome GCA_002238415.1, (C)) both superimposed on Cu-MMO from the model methanotroph Methylococcus capsulatus str. Bath (pdb: 3RGB) (green) with a global model quality estimate of 0.7, and 0.62, respectively, and a quaternary structure quality score of 0.57, and 0.55, respectively.

Figure S2. Formaldehyde oxidation and assimilation capabilities encoded by Binatota genomes. (A) Heatmap of the distribution of formaldehyde oxidation genes in Binatota genomes from different orders. The heatmap colors (as explained in the key) correspond to the percentage of genomes in each order encoding a homologue of the gene in the column header. Shown are the different routes of formaldehyde oxidation, including the (myco)thiol-dependent formaldehyde dehydrogenase *fadH/mscR* (along with mycothiol biosynthesis genes (*mshABC*)), the H_4F-linked pathway (comprising the genes bifunctional methylene-H_4F dehydrogenase and methenyl-H_4F cyclohydrolase (*folD*), reversible formyl-H_4F ligase (*ftfL*), irreversible formyl-H_4F hydrolase (*purU*)), the glutathione-independent formaldehyde dehydrogenase (*fdhA*), and the glutathione-dependent formaldehyde (comprising the S-(hydroxymethyl)glutathione synthase (*gfa*), NAD- and glutathione-dependent formaldehyde dehydrogenase (*frmA*), S-formylglutathione hydrolase (*frmB*)). Also shown is the distribution of the NAD-dependent formate dehydrogenase (EC: 1.17.1.9) (*fdh*) for formate oxidation. (B) Overview of the pathways for formaldehyde assimilation via the serine cycle (left), and glyoxylate regeneration via the ethylmalonyl-CoA pathway and the glyoxylate shunt (GS) (right). Names of enzymes are shown in red and their distribution in the Binatota genomes from different orders is shown in the heatmap in (C). *glyA*, glycine hydroxymethyltransferase [EC:2.1.2.1]; *sgaA*; serine-glyoxylate transaminase [EC:2.6.1.45]; *hprA*, glycerate dehydrogenase [EC:1.1.1.29]; *gck*, glycerate 2-kinase [EC:2.7.1.165]; *ppc*, phosphoenolpyruvate carboxylase [EC:4.1.1.31]; *pckA*, phosphoenolpyruvate carboxykinase; *mdh*, malate dehydrogenase [EC:1.1.1.37]; *mtkA/B*, malate-CoA ligase [EC:6.2.1.9]; *mcl*, malyl-CoA/(S)-citramalyl-CoA lyase [EC:4.1.3.24 4.1.3.25]; *aceA*, isocitrate lyase [EC:4.1.3.1]; *aceB*, malate synthase [EC:2.3.3.9]; *phbB*, acetoacetyl-CoA reductase [EC:1.1.1.36]; *croR*, 3-hydroxybutyryl-CoA dehydratase [EC:4.2.1.55]; *ccr*, crotonyl-

CoA carboxylase/reductase [EC:1.3.1.85]; *epi*, methylmalonyl-CoA/ethylmalonyl-CoA epimerase [EC:5.1.99.1]; *ecm*, ethylmalonyl-CoA mutase [EC:5.4.99.63]; *mcd*, (2S)-methylsuccinyl-CoA dehydrogenase [EC:1.3.8.12]; *mch*, 2-methylfumaryl-CoA hydratase [EC:4.2.1.148]; *mut*, methylmalonyl-CoA mutase [EC:5.4.99.2]; *mcmA1/A2*, methylmalonyl-CoA mutase [EC:5.4.99.2]. Abbreviations: PEP, phosphoenol pyruvate; OAA, oxaloacetate.

Figure S3. (A) Heatmap of the distribution of various chain-length fatty acid and haloacid degradation genes in Binatota genomes. The heatmap colors (as explained in the key) correspond to the percentage of genomes in each order encoding a homologue of the gene in the column header. (B) Propionyl-CoA degradation pathways encoded by the Binatota genomes. The methylmalonyl CoA (MMCoA) pathway is shown in blue, while the 2-methylcitrate pathway is shown in green. In some genomes, the MMCoA pathway seems to be functional but with a slight modification (shown in purple) that includes glyoxylate assimilation and regeneration. *pmoABC,* Copper membrane monooxygenase with denoting subunits A, B, and C; *prmABC*, propane 2-monooxygenase [EC:1.14.13.227]; *alkB*, alkane 1-monooxygenase [EC:1.14.15.3]; cyp153, Cytochrome P450 alkane hydroxylase [EC 1.14.15.1]; *ladA*, long-chain alkane monooxygenase [EC:1.14.14.28]; *dhaA*, haloalkane dehalogenase [EC:3.8.1.5]; *adh*, alcohol dehydrogenase [EC:1.1.1.1]; EC:1.1.1.80, isopropanol dehydrogenase (NADP+) [EC:1.1.1.80]; *acmA*, acetone monooxygenase (methyl acetate-forming) [EC:1.14.13.226]; *acmB*, methyl acetate hydrolase [EC:3.1.1.114]; EC:1.2.1.3, aldehyde dehydrogenase (NAD+) [EC:1.2.1.3]; E1.2.1.10, acetaldehyde dehydrogenase (acetylating) [EC:1.2.1.10]; *acdAB*, acetate---CoA ligase (ADP-forming) [EC:6.2.1.13]; acs, acetyl-CoA synthase [EC:2.3.1.169]; *atoAD*, acetate CoA/acetoacetate CoA-transferase [EC:2.8.3.8 2.8.3.9]; EC:6.2.1.2, medium-chain acyl-CoA synthetase [EC:6.2.1.2]; *fadD*, long-chain acyl-CoA synthetase [EC:6.2.1.3]; *pccA*, propionyl-CoA carboxylase alpha chain [EC:6.4.1.3]; *epi*, methylmalonyl-CoA/ethylmalonyl-CoA epimerase [EC:5.1.99.1]; *mut*, methylmalonyl-CoA mutase [EC:5.4.99.2]; *mcl*, malyl-CoA/(S)-citramalyl-CoA lyase [EC:4.1.3.24 4.1.3.25]; *mch*, 2-methylfumaryl-CoA hydratase [EC:4.2.1.148]; *mct*, 2-methylfumaryl-CoA isomerase [EC:5.4.1.3]; *meh*, 3-methylfumaryl-CoA hydratase [EC:4.2.1.153]; *smtAB*, succinyl-CoA:(S)-malate CoA-transferase subunit A

[EC:2.8.3.22]; *prpB*, methylisocitrate lyase [EC:4.1.3.30]; *prpC*, 2-methylcitrate synthase [EC:2.3.3.5]; *prpD*, 2-methylcitrate dehydratase [EC:4.2.1.79]; *bcd*, butyryl-CoA dehydrogenase [EC:1.3.8.1]; *acd*, acyl-CoA dehydrogenase [EC:1.3.8.7]; *paaF*, enoyl-CoA hydratase [EC:4.2.1.17]; *crt*, enoyl-CoA hydratase [EC:4.2.1.17]; *paaH*, 3-hydroxybutyryl-CoA dehydrogenase [EC:1.1.1.157]; *phbB*, acetoacetyl-CoA reductase [EC:1.1.1.36]; *atoB*, acetyl-CoA C-acetyltransferase [EC:2.3.1.9]; *fadJ*, 3-hydroxyacyl-CoA dehydrogenase / enoyl-CoA hydratase / 3-hydroxybutyryl-CoA epimerase [EC:1.1.1.35 4.2.1.17 5.1.2.3]; *fadA*, acetyl-CoA acyltransferase [EC:2.3.1.16]; *dehH*, 2-haloacid dehalogenase [EC:3.8.1.2]; EC:3.8.1.3, haloacetate dehalogenase [EC:3.8.1.3]; *glcDEF*, glycolate oxidase [EC:1.1.3.15]; EC:1.1.3.15, (S)-2-hydroxy-acid oxidase [EC:1.1.3.15].

Figure S4. Electron transport chain in the Binatota. (A) Heatmap of the distribution of electron transport chain components in the Binatota genomes and electrons entry points from various substrates. The heatmap colors (as explained in the key) correspond to the percentage of genomes in each order encoding a homologue of the gene in the column header. All subunits of complexes I (NADH-quinone oxidoreductase [EC:7.1.1.2]), and II (succinate dehydrogenase /fumarate reductase [EC:1.3.5.1 1.3.5.4]) were encoded in all genomes but are shown here as single components for ease of visualization. Genes encoding quinone-cytochrome C reductase activities belonged to either complex III (cytochrome bc1; ISP/*cytb*/cyt1) and/or alternate cytochrome III (ACIII; *actABCDEF*), while genes encoding cytochrome c oxidase activities (complex IV) belonged to different families including family A (cytochrome c oxidase aa3; *coxABC*), family C (cytochrome c oxidase cbb3; *ccoNOP*), and/or cytochrome *bd* (*cydAB*). Possible electron transfer proteins between complex III (or alternate complex III) and complex IV belonging to different cytochrome c families are shown. Also shown in (A) is the distribution of the three subunits of the type I respiratory O_2-tolerant H_2-uptake [NiFe] hydrogenase (*hyaABC*) in Binatota genomes. (B) Maximum likelihood phylogenetic tree showing the classification of the *hyaA* genes encoded by the Binatota genomes (magenta) in relation to other [Ni-Fe] hydrogenases. The [Fe-Fe] hydrogenase of *Methanobacterium formicum* was used as the outgroup. Bootstrap support (from 100 bootstraps) is shown for branches with >50% support.

Figure S5. (A) Maximum likelihood phylogenetic tree based on the 16S rRNA gene representatives from six Binatota orders with representative hit sequences (number of sequences in parentheses following the order name) from the IMG and NCBI nt databases identified by Blastn. Orders are color coded following the color scheme in Figure 1, and the number of hits from each database are shown in parentheses. Bootstrap value (from 100 bootstraps) are shown for branches with >70% support. (B-G) Ecological distribution of Binatota-affiliated 16S rRNA sequences. Representative 16S rRNA gene sequences from six out of the seven Binatota orders (order UBA12015 genome assembly did not contain a16S rRNA gene) were searched against Integrated Microbial Genomes & Microbiomes (IMG/M) 16S rRNA public assembled metagenomes database using Blastn and the criteria specified in Materials and Methods. Binatota orders are shown on the X-axis, while percentage abundance in different environments (classified based on the GOLD ecosystem classification scheme) are shown on the Y-axis (B). Further sub-classifications for each environment are shown for (C) terrestrial, (D) freshwater, (E) marine, (F) host-associated, and (G) engineered environments. Details including GenBank accession number of hit sequences are shown in Extended data 3.

Data availability. Genomic bins, predicted proteins, and extended data for Figures 2-3, 5-6, S2-S4, and for Figures 7a-f and S5b-g are available at https://github.com/ChelseaMurphy/Binatota. Maximum likelihood trees (Figure 1 and Figure S5a) can be accessed at: https://itol.embl.de/shared/1WgxEjrQfEYWk. Maximum likelihood trees for chlorophyll biosynthesis genes are available at https://itol.embl.de/shared/34y3BUHcQd7Lh.

Extended data 1. Different sheets correspond to the heatmaps in Figures 2-3, 5-6 and S2-S4 in the main text. Each sheet references the protein IDs in each of the 108 Binatota genomes corresponding to the gene in the column header. The actual amino acid sequences can be found in the amino acid fasta files available at https://github.com/ChelseaMurphy/Binatota by searching by the protein ID in the corresponding genome file.

Extended data 2. Different sheets correspond to different Binatota orders. Each sheet lists the accession numbers, source, and ecosystem-level classification of the NCBI nt database hits identified using Blastn. These data were used to construct Figure 7.

Extended data 3. Different sheets correspond to different Binatota orders. Each sheet lists the accession numbers, source, and ecosystem-level classification of the IMG database hits identified using Blastn. These data were used to construct Figure S5.

References

1. Lessmeier L, Hoefener M, Wendisch VF. 2013. Formaldehyde degradation in Corynebacterium glutamicum involves acetaldehyde dehydrogenase and mycothiol-dependent formaldehyde dehydrogenase. Microbiology 159:2651-2662.
2. Chistoserdova L. 2011. Modularity of methylotrophy, revisited. Environ Microbiol 13:2603-22.
3. Dubey AA, Wani SR, Jain V. 2018. Methylotrophy in Mycobacteria: Dissection of the methanol metabolism pathway in *Mycobacterium smegmatis*. J Bacteriol 200.
4. Kornberg HL, Krebs HA. 1957. Synthesis of cell constituents from C2-units by a modified tricarboxylic acid cycle. Nature 179:988-91.
5. Alber BE, Spanheimer R, Ebenau-Jehle C, Fuchs G. 2006. Study of an alternate glyoxylate cycle for acetate assimilation by *Rhodobacter sphaeroides*. Mol Microbiol 61:297-309.
6. Chen Q, Janssen DB, Witholt B. 1995. Growth on octane alters the membrane lipid fatty acids of *Pseudomonas oleovorans* due to the induction of alkB and synthesis of octanol. J Bacteriol 177:6894-901.
7. van Beilen JB, Funhoff EG. 2007. Alkane hydroxylases involved in microbial alkane degradation. Appl Microbiol Biotechnol 74:13-21.
8. Li L, Liu X, Yang W, Xu F, Wang W, Feng L, Bartlam M, Wang L, Rao Z. 2008. Crystal structure of long-chain alkane monooxygenase (LadA) in complex with coenzyme FMN: unveiling the long-chain alkane hydroxylase. J Mol Biol 376:453-65.
9. Nagata Y, Miyauchi K, Damborsky J, Manova K, Ansorgova A, Takagi M. 1997. Purification and characterization of a haloalkane dehalogenase of a new substrate class from a gamma-hexachlorocyclohexane-degrading bacterium, *Sphingomonas paucimobilis* UT26. Appl Environ Microbiol 63:3707-10.
10. Sun C, Benlekbir S, Venkatakrishnan P, Wang Y, Hong S, Hosler J, Tajkhorshid E, Rubinstein JL, Gennis RB. 2018. Structure of the alternative complex III in a supercomplex with cytochrome oxidase. Nature 557:123-126.
11. Choi DW, Kunz RC, Boyd ES, Semrau JD, Antholine WE, Han JI, Zahn JA, Boyd JM, de la Mora AM, DiSpirito AA. 2003. The membrane-associated methane monooxygenase (pMMO) and pMMO-NADH:quinone oxidoreductase complex from *Methylococcus capsulatus* Bath. J Bacteriol 185:5755-64.
12. Nguyen HH, Elliott SJ, Yip JH, Chan SI. 1998. The particulate methane monooxygenase from *Methylococcus capsulatus* (Bath) is a novel copper-containing three-subunit enzyme. Isolation and characterization. J Biol Chem 273:7957-66.
13. Wulff P, Day CC, Sargent F, Armstrong FA. 2014. How oxygen reacts with oxygen-tolerant respiratory [NiFe]-hydrogenases. Proc Natl Acad Sci USA 111:6606-11.
14. Sargent F. 2016. The Model [NiFe]-Hydrogenases of *Escherichia coli*. Adv Microb Physiol 68:433-507.
15. Volbeda A, Darnault C, Parkin A, Sargent F, Armstrong FA, Fontecilla-Camps JC. 2013. Crystal structure of the O(2)-tolerant membrane-bound hydrogenase 1 from *Escherichia coli* in complex with its cognate cytochrome b. Structure 21:184-190.
16. Carere CR, Hards K, Houghton KM, Power JF, McDonald B, Collet C, Gapes DJ, Sparling R, Boyd ES, Cook GM, Greening C, Stott MB. 2017. Mixotrophy drives niche expansion of verrucomicrobial methanotrophs. ISME J 11:2599-2610.

17. Anantharaman K, Brown CT, Hug LA, Sharon I, Castelle CJ, Probst AJ, Thomas BC, Singh A, Wilkins MJ, Karaoz U, Brodie EL, Williams KH, Hubbard SS, Banfield JF. 2016. Thousands of microbial genomes shed light on interconnected biogeochemical processes in an aquifer system. Nat Commun 7:13219.
18. Anantharaman K, Duhaime MB, Breier JA, Wendt KA, Toner BM, Dick GJ. 2014. Sulfur oxidation genes in diverse deep-sea viruses. Science 344:757-60.
19. Hausmann B, Pelikan C, Herbold CW, Köstlbacher S, Albertsen M, Eichorst SA, Glavina Del Rio T, Huemer M, Nielsen PH, Rattei T, Stingl U, Tringe SG, Trojan D, Wentrup C, Woebken D, Pester M, Loy A. 2018. Peatland Acidobacteria with a dissimilatory sulfur metabolism. ISME J 12:1729-1742.
20. Kato S, Sakai S, Hirai M, Tasumi E, Nishizawa M, Suzuki K, Takai K. 2018. Long-term cultivation and metagenomics reveal ecophysiology of previously uncultivated thermophiles involved in biogeochemical nitrogen cycle. Microbes Environ 33:107-110.
21. Lawson CE, Wu S, Bhattacharjee AS, Hamilton JJ, McMahon KD, Goel R, Noguera DR. 2017. Metabolic network analysis reveals microbial community interactions in anammox granules. Nat Commun 8:15416.
22. Parks DH, Chuvochina M, Waite DW, Rinke C, Skarshewski A, Chaumeil PA, Hugenholtz P. 2018. A standardized bacterial taxonomy based on genome phylogeny substantially revises the tree of life. Nat Biotechnol 36:996-1004.
23. Parks DH, Rinke C, Chuvochina M, Chaumeil PA, Woodcroft BJ, Evans PN, Hugenholtz P, Tyson GW. 2017. Recovery of nearly 8,000 metagenome-assembled genomes substantially expands the tree of life. Nat Microbiol 2:1533-1542.
24. Slaby BM, Hackl T, Horn H, Bayer K, Hentschel U. 2017. Metagenomic binning of a marine sponge microbiome reveals unity in defense but metabolic specialization. ISME J 11:2465-2478.
25. Sorensen JW, Dunivin TK, Tobin TC, Shade A. 2019. Ecological selection for small microbial genomes along a temperate-to-thermal soil gradient. Nat Microbiol 4:55-61.
26. Yao Q, Li Z, Song Y, Wright SJ, Guo X, Tringe SG, Tfaily MM, Paša-Tolić L, Hazen TC, Turner BL, Mayes MA, Pan C. 2018. Community proteogenomics reveals the systemic impact of phosphorus availability on microbial functions in tropical soil. Nat Ecol Evol 2:499-509.
27. Graham EB, Crump AR, Kennedy DW, Arntzen E, Fansler S, Purvine SO, Nicora CD, Nelson W, Tfaily MM, Stegen JC. 2018. Multi 'omics comparison reveals metabolome biochemistry, not microbiome composition or gene expression, corresponds to elevated biogeochemical function in the hyporheic zone. Sci Total Environ 642:742-753.
28. Tran PQ, McIntyre PB, Kraemer BM, Vadeboncoeur Y, Kimirei IA, Tamatamah R, McMahon KD, Anantharaman K. 2019. Depth-discrete eco-genomics of Lake Tanganyika reveals roles of diverse microbes, including candidate phyla, in tropical freshwater nutrient cycling. bioRxiv doi:10.1101/834861:834861.
29. Thomas SC, Tamadonfar KO, Seymour CO, Lai D, Dodsworth JA, Murugapiran SK, Eloe-Fadrosh EA, Dijkstra P, Hedlund BP. 2019. Position-specific metabolic probing and metagenomics of microbial communities reveal conserved central carbon metabolic network activities at high temperatures. Front Microbiol 10:1427.
