# Supplementary figures and images for: Genomic Analysis of the Yet-Uncultured Binatota Reveals Broad Methylotrophic, Alkane-Degradation, and Pigment Production Capacities
Source: mBio. 2021 May 18;12(3):e00985-21. doi: 10.1128/mBio.00985-21 (PMC8262859; doi:10.1128/mBio.00985-21)

### Figure S2

A

[illegible]

B

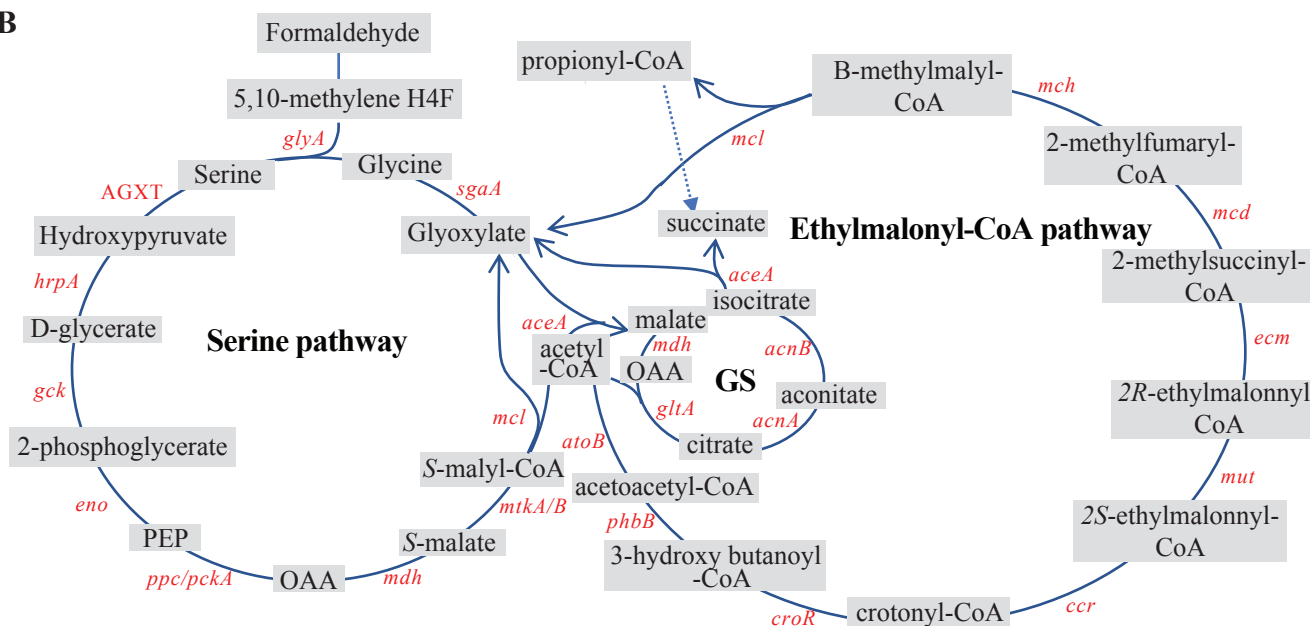

**C**

[illegible]

Supplement: FIG S2 [file mbio.00985-21-sf002.pdf]

### Figure S4

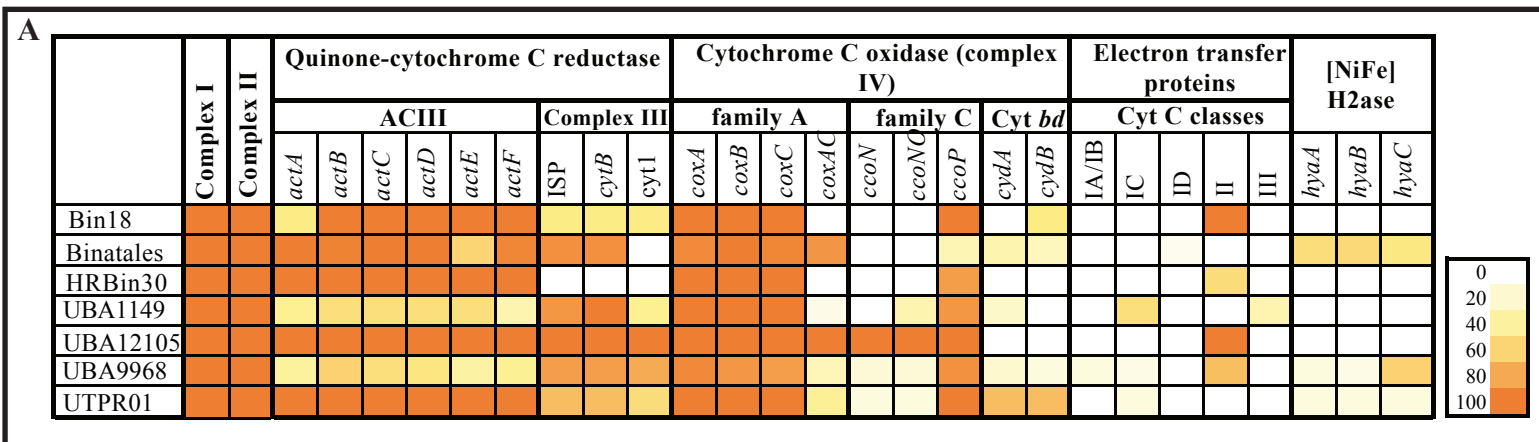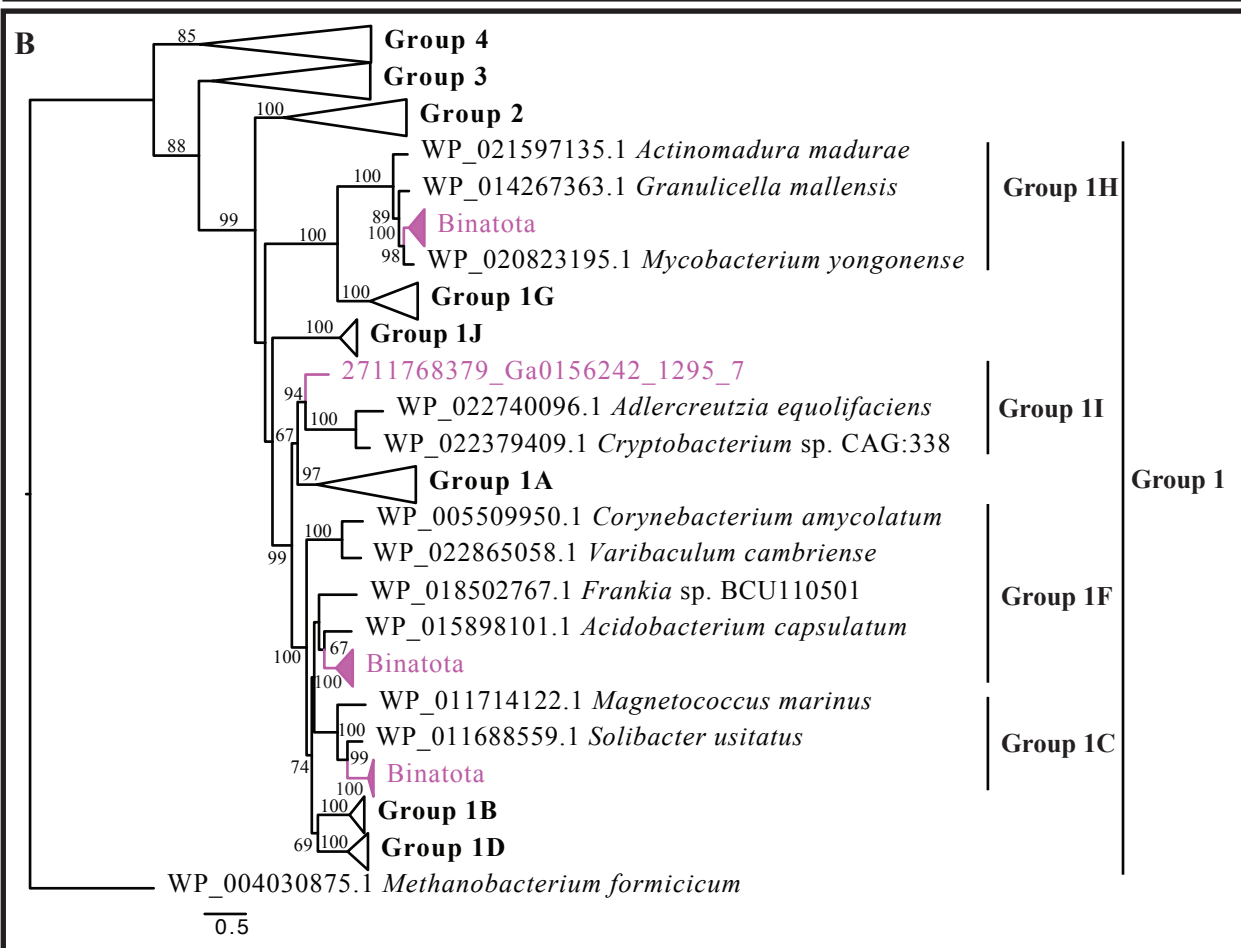

Supplement: FIG S4 [file mbio.00985-21-sf004.pdf]

Figure S5

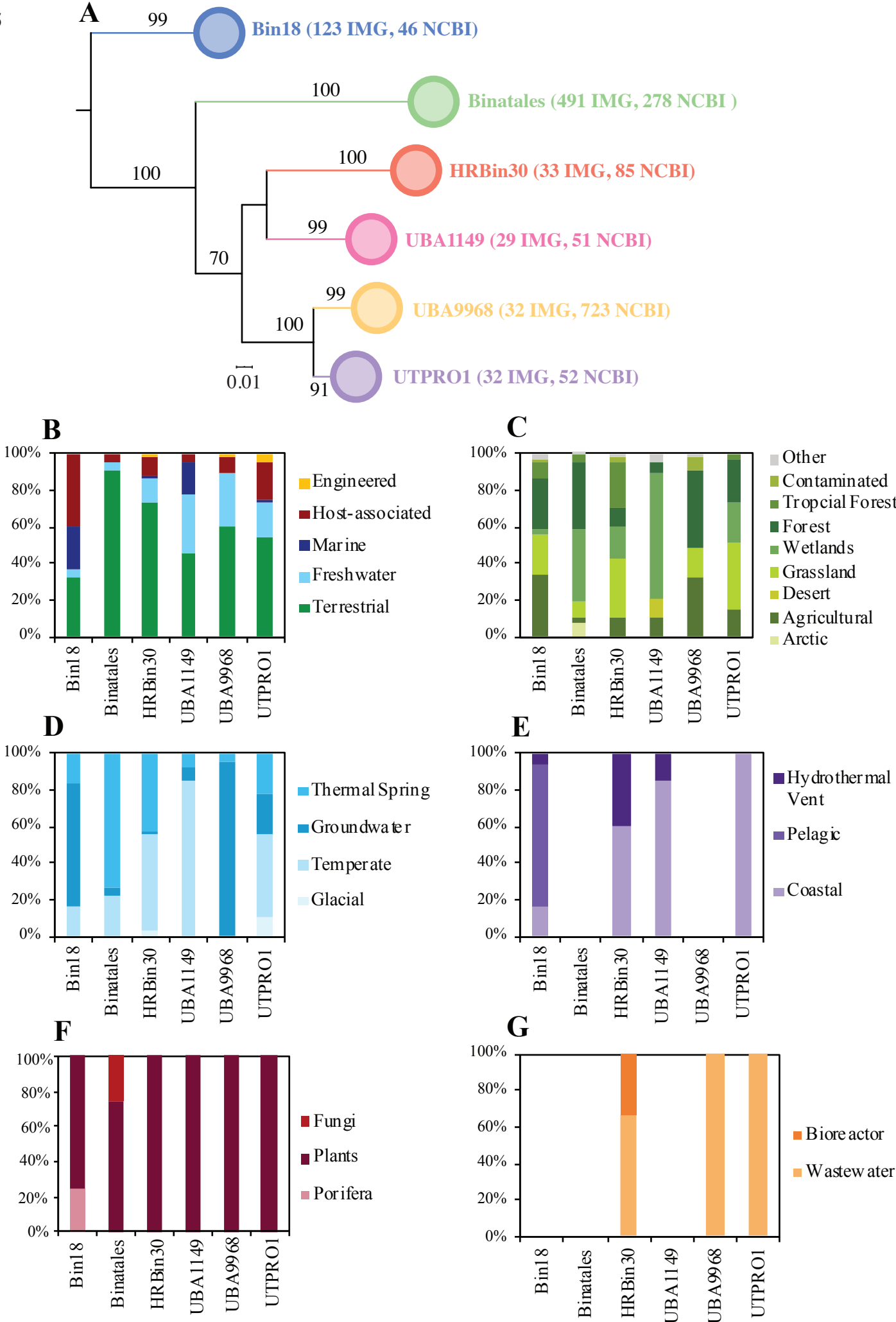

Supplement: FIG S5 [file mbio.00985-21-sf005.pdf]
