# Supplementary material for: Genomic Analysis of the Yet-Uncultured Binatota Reveals Broad Methylotrophic, Alkane-Degradation, and Pigment Production Capacities
Source: mBio. 2021 May 18;12(3):e00985-21. doi: 10.1128/mBio.00985-21 (PMC8262859; doi:10.1128/mBio.00985-21)
Supplement: FIG S3 [file mbio.00985-21-sf003.pdf]

Figure S3

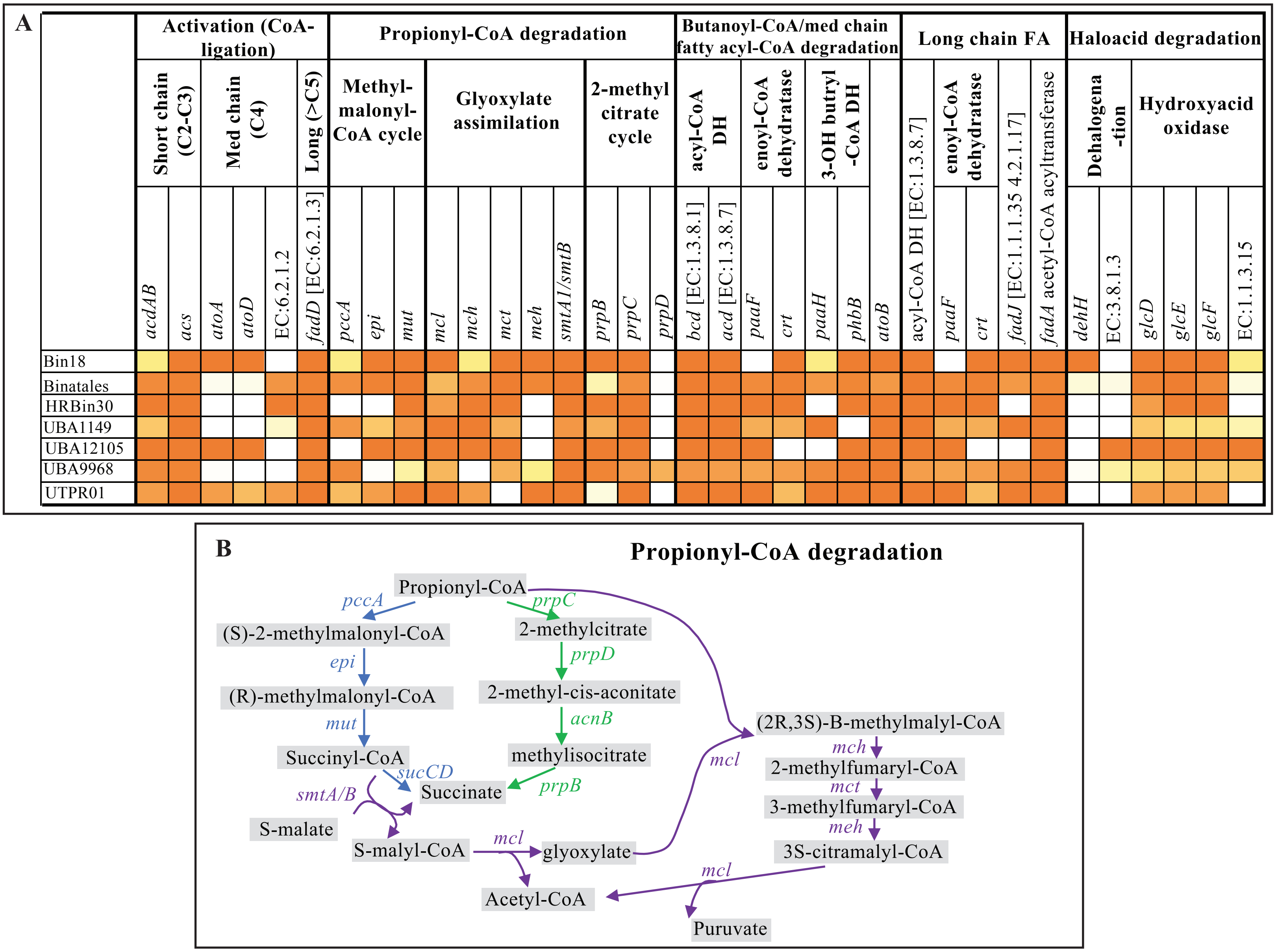
A
Activation (CoA-ligation)
Propionyl-CoA degradation
Butanoyl-CoA/med chain fatty acyl-CoA degradation
Long chain FA
Haloacid degradation
Short chain (C2-C3)
Med chain (C4)
Long (>C5)
Methyl-malonyl-CoA cycle
Glyoxylate assimilation
2-methyl citrate cycle
acyl-CoA DH
enoyl-CoA dehydratase
3-OH butryl -CoA DH
enoyl-CoA dehydratase
Dehalogena -tion
Hydroxyacid oxidase
acdAB
acs
atoA
atoD
EC:6.2.1.2
fadD [EC:6.2.1.3]
pccA
epi
mut
mcl
mch
mct
meh
smtA1/smtB
prpB
prpC
prpD
bcd [EC:1.3.8.1]
acd [EC:1.3.8.7]
paaF
crt
paaH
phbB
atoB
acyl-CoA DH [EC:1.3.8.7]
paaF
crt
fadJ [EC:1.1.1.35 4.2.1.17]
fadA acetyl-CoA acyltransferase
dehH
EC:3.8.1.3
glcD
glcE
glcF
EC:1.1.3.15
Bin18
Binatales
HRBin30
UBA1149
UBA12105
UBA9968
UTPR01
B
Propionyl-CoA degradation
Propionyl-CoA
pccA
prpC
(S)-2-methylmalonyl-CoA
2-methylcitrate
epi
prpD
(R)-methylmalonyl-CoA
2-methyl-cis-aconitate
mut
acnB
(2R,3S)-B-methylmalyl-CoA
Succinyl-CoA
methylisocitrate
mch
mcl
2-methylfumaryl-CoA
sucCD
mct
smtA/B
Succinate
prpB
3-methylfumaryl-CoA
meh
S-malate
mcl
S-malyl-CoA
glyoxylate
3S-citramalyl-CoA
mcl
Acetyl-CoA
Puruvate
